# Large Language Models in Medical Diagnostics: Scoping Review With Bibliometric Analysis

**DOI:** 10.2196/72062

**Published:** 2025-06-09

**Authors:** Hankun Su, Yuanyuan Sun, Ruiting Li, Aozhe Zhang, Yuemeng Yang, Fen Xiao, Zhiying Duan, Jingjing Chen, Qin Hu, Tianli Yang, Bin Xu, Qiong Zhang, Jing Zhao, Yanping Li, Hui Li

**Affiliations:** 1 Department of Reproductive Medicine Xiangya Hospital Central South University Changsha China; 2 Clinical Research Center for Women's Reproductive Health in Hunan Province Changsha China; 3 Xiangya School of Medicine Central South University Changsha China; 4 School of Biomedical Sciences and Engineering South China University of Technology Guangzhou China; 5 Department of Metabolism and Endocrinology Second Xiangya Hospital of Central South University Changsha China

**Keywords:** large language model, scoping review, medical diagnosis, bibliometric analysis, artificial intelligence

## Abstract

**Background:**

The integration of large language models (LLMs) into medical diagnostics has garnered substantial attention due to their potential to enhance diagnostic accuracy, streamline clinical workflows, and address health care disparities. However, the rapid evolution of LLM research necessitates a comprehensive synthesis of their applications, challenges, and future directions.

**Objective:**

This scoping review aimed to provide an overview of the current state of research regarding the use of LLMs in medical diagnostics. The study sought to answer four primary subquestions, as follows: (1) Which LLMs are commonly used? (2) How are LLMs assessed in diagnosis? (3) What is the current performance of LLMs in diagnosing diseases? (4) Which medical domains are investigating the application of LLMs?

**Methods:**

This scoping review was conducted according to the Joanna Briggs Institute Manual for Evidence Synthesis and adheres to the PRISMA-ScR (Preferred Reporting Items for Systematic Reviews and Meta-Analyses extension for Scoping Reviews). Relevant literature was searched from the Web of Science, PubMed, Embase, IEEE Xplore, and ACM Digital Library databases from 2022 to 2025. Articles were screened and selected based on predefined inclusion and exclusion criteria. Bibliometric analysis was performed using VOSviewer to identify major research clusters and trends. Data extraction included details on LLM types, application domains, and performance metrics.

**Results:**

The field is rapidly expanding, with a surge in publications after 2023. GPT-4 and its variants dominated research (70/95, 74% of studies), followed by GPT-3.5 (34/95, 36%). Key applications included disease classification (text or image-based), medical question answering, and diagnostic content generation. LLMs demonstrated high accuracy in specialties like radiology, psychiatry, and neurology but exhibited biases in race, gender, and cost predictions. Ethical concerns, including privacy risks and model hallucination, alongside regulatory fragmentation, were critical barriers to clinical adoption.

**Conclusions:**

LLMs hold transformative potential for medical diagnostics but require rigorous validation, bias mitigation, and multimodal integration to address real-world complexities. Future research should prioritize explainable artificial intelligence frameworks, specialty-specific optimization, and international regulatory harmonization to ensure equitable and safe clinical deployment.

## Introduction

In the critical domain of health care, the efficacy of medical decision-making and diagnostic accuracy is essential for managing medical conditions effectively. To bolster these processes, artificial intelligence (AI) models have been increasingly used, demonstrating the potential to rival the diagnostic prowess of seasoned clinicians [1,2].

Large language models (LLMs) are sophisticated AI systems that undergo extensive pretraining, absorbing statistical laws and discernible patterns from extensive datasets [3]. Consequently, they possess the remarkable ability to autonomously generate responses to inquiries and engage in interactive dialogues with users [4]. This capability has also raised the interest of the medical community, who see in LLMs a tool that could significantly enhance various facets of health care, especially in diagnostics [5]. LLMs have presented promising performance in undertaking medical tasks [4,6]; for instance, models such as OpenAI’s ChatGPT and Google’s Bard have showcased the potential to enhance clinical decision-making processes [7], refine diagnostic accuracy [8], and facilitate the synthesis of complex medical literature [9].

Despite the growing interest in LLMs in diagnostic application, there is a noticeable absence of comprehensive analysis that examines the evolution and analytic appraisal of LLMs in medical diagnosis. Current scoping reviews on LLMs are predominantly conceptual and focused mainly on the entire area of biomedical health [10], highlighting an urgent need for a targeted, domain-specific review to guide future research directions [4,11,12]. With this scoping review, we therefore intend to answer the following research question (RQ): What is the state of research regarding medical diagnosis based on LLMs?

However, traditional scoping review alone may struggle to capture the rapid expansion of LLM research, which has grown exponentially in volume and complexity. To address this, we integrated bibliometric analysis—a quantitative and visualized method for evaluating research landscape and trend-within the scoping review [13]. This hybrid approach enables a dual perspective: the bibliometric analysis serves as an indicator for scoping review, mapping the fields growth, interest, and future trend, while the scoping reviews dig deeper into synthesizing qualitative and quantitative insights on LLMs’ diagnostic applications. Together, the hybrid approach provides a holistic view of the state of research regarding medical diagnosis. Therefore, complemented by bibliometric clustering, we broke the large research question into 4 smaller subjects, each representing a major research focus that has been extensively studied in the field, as follows:

1. Which LLMs are commonly used in medical diagnosis?
2. How to examine the performance of LLM in medical diagnosis?
3. What is the current performance of LLMs in diagnosing diseases?
4. Which medical domains are investigating the application of LLMs?

With a combination of scoping review and bibliometric method, we hope to enhance readers’ comprehension of LLMs and provide a guideline for prospective collaborative pursuits and clinical implementations.

## Methods

### Overview

This scoping review was conducted according to the Joanna Briggs Institute Manual for Evidence Synthesis and adheres to the PRISMA-ScR (Preferred Reporting Items for Systematic Reviews and Meta-Analyses extension for Scoping Reviews) [14].

### Search Strategy and Study Selection

Web of Science, PubMed, Embase, IEEE Xplore, and ACM Digital Library databases were searched as the source of relevant literature from 2022 to January 2025. The first search was conducted on September 27, 2024, and the final search on January 12, 2025. Multimedia Appendix 1 provides the detailed search strategies. Raw data were first imported into the Rayyan platform. Subsequently, 4 authors (HS, YS, AZ, and YY) independently screened for potentially relevant articles in a blind mode with the following procedure: duplicates were first removed, followed by a screening of browsing titles and abstracts. The remaining articles were screened for the second time to evaluate the full text of all eligible articles. At any point in the process, conflicting articles were resolved by RL. The eligibility criteria are listed in subsequent section.

### Inclusion and Exclusion Criteria

The inclusion criteria were as follows: (1) articles that focused on reporting the outcome of LLMs in medical diagnosis–related field and (2) articles that described the application of LLMs for medical diagnosis. LLMs here are defined as deep learning models with more than one million parameters, trained on unlabeled text data

To obtain only necessary data, we excluded articles that were conference abstracts or abstracts only and (2) comments and retracted articles.

### Bibliometric Analysis

After the selection of included articles, the data from the included articles from Web of Science were first exported in tab-delimited format and then imported into Microsoft Excel 2019 for preliminary collation. Non–Web of Science articles were manually entered into Microsoft Excel 2019 by 2 independent researchers (AZ and RL) to ensure accuracy. The combined dataset was converted to tab-delimited format for compatibility with a bibliometric tool. VoSviewer (Leiden University) was used to map research trends and frontiers in LLM applications for medical diagnosis. This analysis identified 3 dominant research clusters reflecting key thematic foci, which subsequently informed the design of data extraction categories. The bibliometric analysis objectively identified field-level hot spots and trends, while the scoping review contextualized these patterns through qualitative appraisal of study designs and clinical validations. This dual approach ensured both macroscopic trend detection (via VOSviewer keyword clustering) and microscopic evidence evaluation (via structured data extraction).

### Data Extraction

The bibliometric clusters guided the development of an extraction template to systematically capture bibliometric attributes (eg, authors, publication years, and countries), thematic focus (eg, specialty task and LLM type), and clinical validation metrics (eg, primary end point, comparator, and primary result). Two authors (HS and YS) independently extracted data using this template, resolving discrepancies through consensus discussions. A pilot extraction on 10% of articles preceded full-text review to refine category definitions.

## Results

### Study Selection and Characteristics

**Figure 1:**
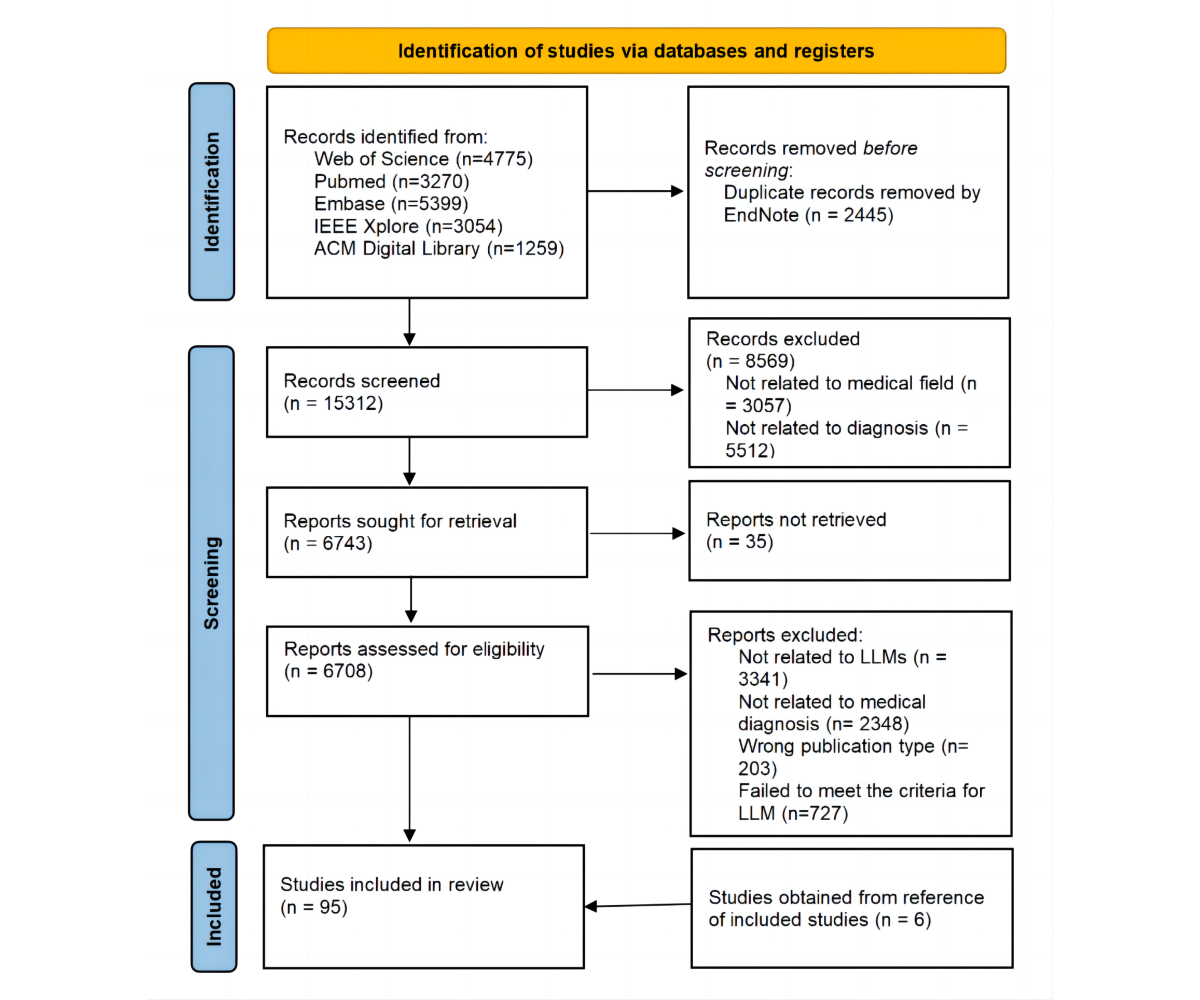
Illustration of study selection process. LLM: large language model.

**Figure 2:**
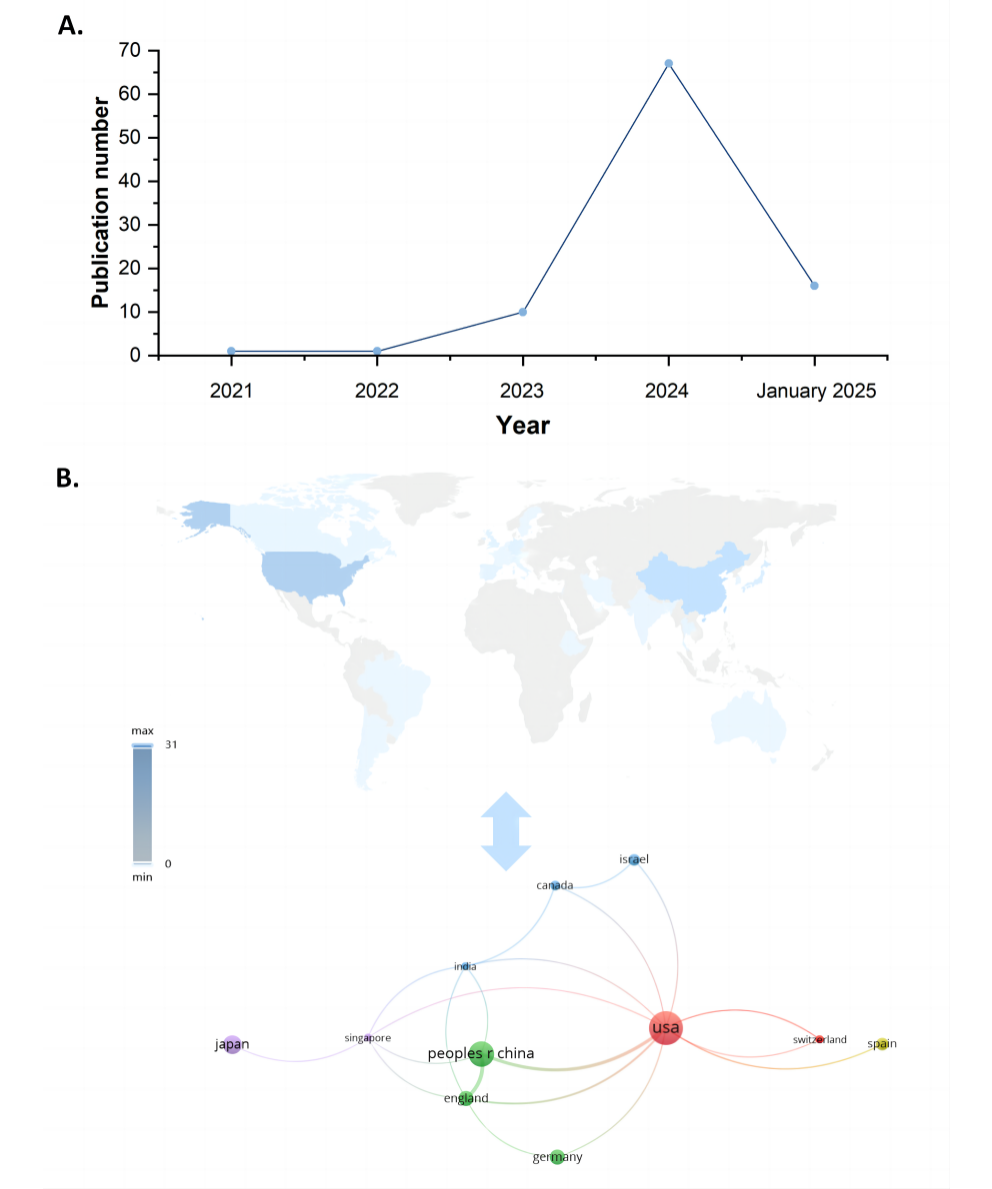
(A) Study distribution by final publication year and (B) study distribution across countries and bibliometric visualization of country collaborations.

Figure 1 displays the study selection process. A total of 17,757 records were identified from Web of Science, PubMed, Embase, IEEE Xplore, and ACM Digital Library databases. After removing 2445 duplicates, we screened titles and abstracts, and 6708 records were assessed for inclusion, resulting in 95 articles in the final review. The overall characteristic of included articles is provided in Multimedia Appendix 2 [15-108]. Figure 2A illustrates the distribution of articles by year, which indicates that this research area is still emerging, with a significant increase in publications occurring in 2024. This trend suggests that the field is likely to continue experiencing positive growth over the coming years. Figure 2B illustrates that the United States and China are the most productive countries in the research of LLM in diagnosis. In addition, the United States has the highest number of interconnected targets in country cooperation. It is suggested for other countries’ researchers to strengthen international cooperation and communication for this research topic.

### Bibliometric Analysis of Articles

**Figure 3:**
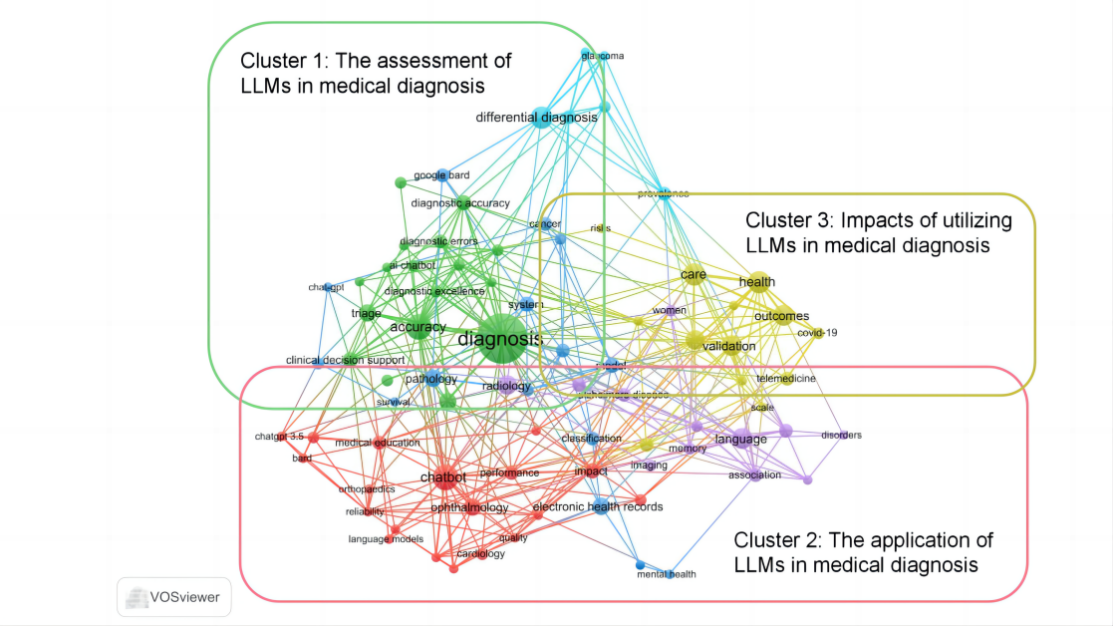
Bibliometric labeling of articles’ keywords. LLM: large language model.

We used VOSviewer software for bibliometric labeling of included studies, and a total of 65 keywords and 3 clusters were obtained (Figure 3). Cluster 1, “The assessment of LLMs in medical diagnosis,” is noted in green, with keywords including “accuracy” and “diagnostic accuracy.” This cluster focuses on the accuracy and reliability of LLMs in medical diagnosis and how they support clinical decision-making and triage. Cluster 2, “The application of LLMs in medical diagnosis,” is represented by red, with keywords including “orthopedics,” “cardiology,” and “ophthalmology,” among others. This cluster illustrates the application of LLMs in diagnosis across various medical specialties, including orthopedics, cardiology, and ophthalmology. Cluster 3, “Impacts of using LLMs in medical diagnosis,” is indicated in yellow, with keywords including “health,” and “outcomes,” among others. This cluster focuses on the practical outcomes and impacts of using LLMs in medical diagnosis, including their impact on care, health outcomes, and telemedicine. These clusters are in line with other reported analysis and reviews, suggesting a high reliability [109,110].

We subsequently labeled the articles into the 3 clusters (overlapping articles remained in all mother clusters) for further analysis. Results are provided in Multimedia Appendix 2.

### Quantitative Analysis of LLM Models Used in Current Research

As shown in Table 1, among the 95 included articles, GPT-4 and its variant models were studied in 70 (74%) studies, followed by GPT-3.5 and its variant models in 34 (36%) studies, specific developed GPTs in 9 (9%) studies, Claude and its variant models in 9 (9%) studies, Gemini and its variant models in 8 (8%) studies, and Llama and its variant models in 8 (8%) of the included studies. This suggests that ChatGPT is currently the most popular LLM used in medical diagnosis.

It is also worth noting that specifically developed GPT versions have also surged in numbers in recent years, indicating a translation from general to specific LLMs. Among the specifically developed GPT versions, we extracted 3 major paths for developing a specific GPT version, as described in Table 2 and Textbox 1.

**Table 1:** Qualitative analysis of large language models (LLMs) used in studies.

LLMs	Frequency, n
ChatGPT-4 (variant model included)	70
ChatGPT-3 (variant model included)	34
Specific developed GPT	9
Gemini	8
Llama	8
Claude	9
Others	5

**Table 2:** Quantitative analysis of ways of developing specific GPT versions in studies.

Ways of developing specifically developed GPT versions	Frequency, n
Prompt tuning	8
Fine-tuning	7
Pretrained from scratch	2

**Major paths in developing specific GPT versions.**

**Prompt tuning**

- This involves using carefully crafted prompts to guide the model’s output, often without modifying the model’s weights.
- This method is often used to enhance the performance of existing large language models (LLMs) or to mitigate bias in LLMs [15,111,112].

**Fine-tuning**

- This involves using a subset of labeled data to adjust the model’s weights, allowing it to better adapt to a specific task or domain [113].
- A typical example of this type of LLM is Med-PaLM 2, which is trained based on fine-tuning of Google PaLM [16].

**Pretrained from scratch**

- This involves using a large corpus of unlabeled text to train the model from the beginning, learning general language patterns and representations [114].
- A typical example of this type of LLM is BioGPT, which was pretrained on a corpus of PubMed articles from scratch [115].

### Application and Evaluation of LLMs in Diagnosing Diseases

#### Overview

**Figure 4:**
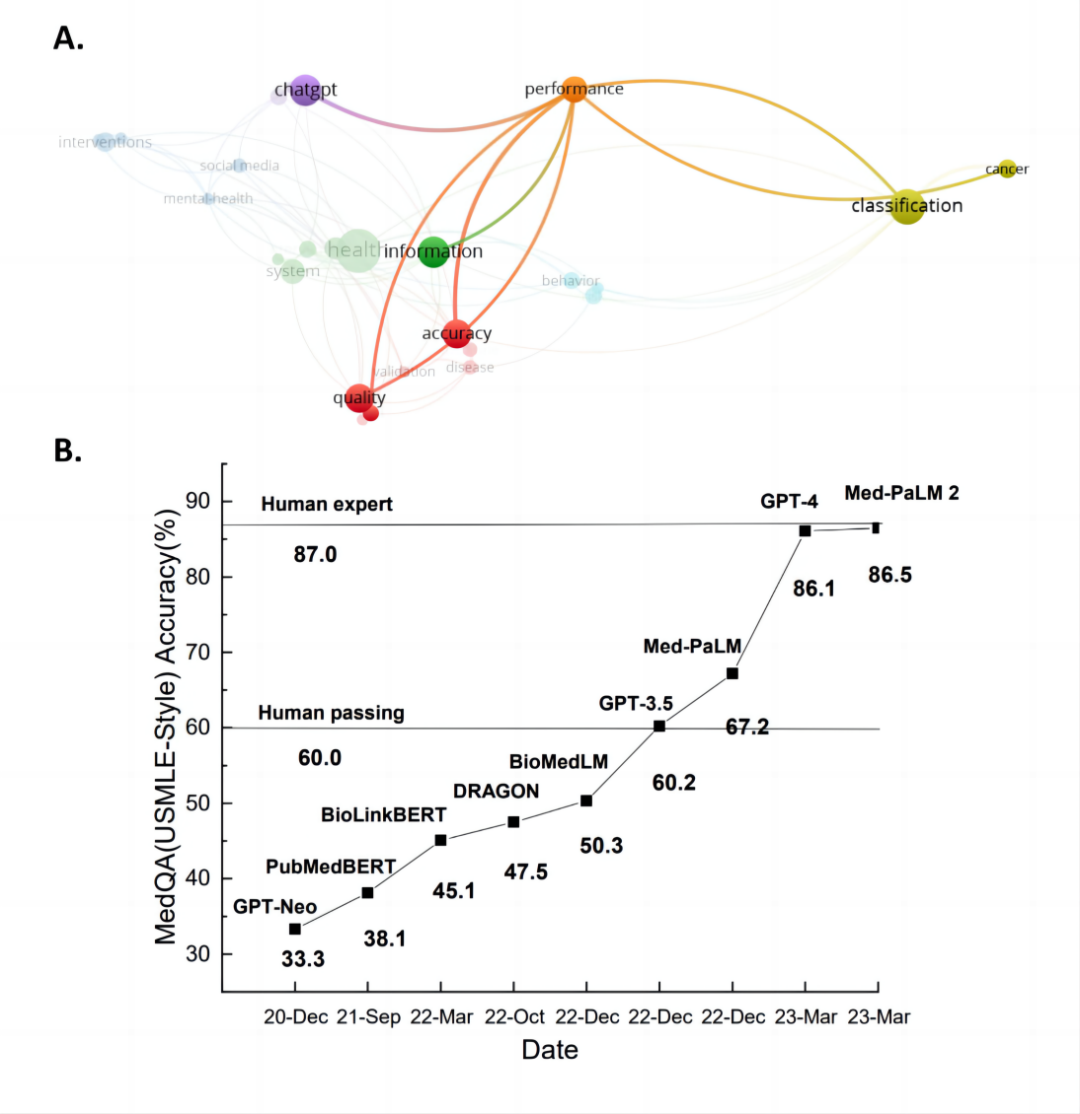
(A) Nested bibliometric analysis of performance evaluation in studies; (B) quantitative analysis of the major assessment aspects; and (C) existing large language models' performance on accuracy. USMLE: United States Medical Licensing Examination.

To assist our understanding of the overall scope of how performance of LLMs were examined, we further conducted a clustering analysis within the articles in cluster 1 and cluster 2 (nested bibliometric analysis). As shown in Figure 4, this suggested that the application and evaluation of LLMs can be mainly categorized into three aspects: (1) disease classification, (2) medical question answering, and (3) quality of generated diagnostic content (Tables 3 and 4).

**Table 3:** Quantitative analysis of the major assessment aspects.

Assessment aspects	Frequency, n
Diagnosis accuracy	77
Disease classification	14
Quality of generated diagnosis content	6

**Table 4:** Detailed description of application.

Performance and evaluation domain and examples			Description		Reference
**Disease classification**					
	Primary and secondary glaucoma	A total of 26 glaucoma cases were selected as a convenience sample; researchers compared the diagnostic performance of GPT-4o with that of ophthalmologists of varying experience levels, focusing on both primary and differential diagnoses of glaucoma.		[17]	
	Melanoma	Prompts were designed to either involve conditioning of asymmetry, border irregularity, color variation, diameter >6 mm, and evolution melanoma features or to assess effects of background skin color on predictions.		[18,116,117]	
**Diagnosis QA^a^**					
	MedQA answering	The MedQA dataset is a QA dataset oriented toward the medical field, emulating the style of the United States Medical Licensing Examination. It contains questions in English, simplified Chinese, and traditional Chinese, aiming to assess the model’s understanding and reasoning abilities in medical knowledge.		[118]	
	PubMedQA answering	The PubMedQA dataset is a novel biomedical QA dataset collected from PubMed abstracts. It requires models to be capable of understanding and reasoning biomedical research texts, especially their quantitative content, to answer research questions.		[119]	
	MedMCQA answering	MedMCQA is a large-scale multiple-choice QA dataset specifically designed to address practical medical entrance examination problems. It contains over 194,000 high-quality multiple-choice questions from AIIMS^b^ and NEET^c^ PG^d^ entrance exams, covering 2400 health care topics and 21 medical subjects.		[120]	
	DSM-5^e^ Clinical Cases book	The book clarifies and discusses psychiatric diagnosis with a particular focus on how diagnoses have evolved from the *DSM-5*. It is commonly used to determine accuracy of mental health diagnosis.		[121]	
	NEJM^f^ image challenge dataset	This dataset includes medical image challenges from the *NEJM*. It is often used to determine accuracy of vision diagnosis.		[122]	
**Quality of generated diagnostic content**					
	Responsiveness	Test to see if LLMs^g^ can respond to every question, instead of giving “I’m sorry, I cannot provide medical diagnoses or interpret medical images.”		[122]	
	Ethical bias	Critically evaluate LLMs in their generated diagnostic content for potential bias in gender and race		[19]	
	Cognitive bias	Use specifically developed QA sets that include clinically biased questions as compared to unbiased ones to test LLMs for their generated diagnostic content.		[15]	

^a^QA: question and answer.

^b^AIIMS: All India Institute of Medical Sciences.

^c^NEET: National Eligibility cum Entrance Test.

^d^PG: postgraduate.

^e^DSM-5: Diagnostic and Statistical Manual of Mental Disorders, Fifth Edition.

^f^NEJM: New England Journal of Medicine.

^g^LLM: large language model.

#### Disease Classification

LLMs such as GPT-4 have shown significant potential in disease classification. Their ability to process and analyze vast amounts of textual data makes them valuable tools for classifying diseases. They can be categorized into text-based classification and image-based classification.

For text-based classification, LLMs have been successfully applied to detect and classify diseases, such as gout and calcium pyrophosphate deposition disease, from electronic health records (EHRs), outperforming traditional methods like regex-based approaches [123]. They achieved high classification accuracy and predictive values, demonstrating their capability to handle non-English medical documents effectively. In addition, in clinicopathological conferences, LLMs such as ChatGPT and Google Bard have been used to generate differential diagnoses for neurodegenerative disorders. While they correctly identified primary diagnoses in a significant number of cases, their inclusion of correct diagnoses in broader lists was even higher, indicating their utility in supporting clinical discussions [124]. All of these have shown LLMs’ potential in analyzing unstructured text (eg, patient symptoms and medical histories) to suggest potential diagnoses, which could be used in public health sections for early detection and classification of diseases to reduce financial and physician’s burden.

In addition to text-based classification, recent research have also explored the possibility of LLMs in classifying diseases based on image data. With the introduction of vision-language models, LLMs exhibit powerful representational learning capabilities, enabling them to comprehend, generate, and process various image data types, showcasing their application potential [125,126]. For example, in dermatology, LLMs are being trained to recognize and classify skin conditions from simple phone pictures, which can assist in early detection of diseases like melanoma. Alternatively, LLMs can be used to interpret professional medical images in radiology, such as X-rays [127,128] and MRIs [129]. These LLMs are being trained to identify abnormalities and assist in diagnosing conditions. The integration of LLMs in these areas offers several potential benefits. It can enhance the speed and accuracy of diagnosis, reduce the workload on health care professionals, and provide consistent interpretations that can be especially valuable in remote or underserved areas where access to specialized care is limited. Furthermore, LLMs can be trained to recognize subtle patterns and features that may be challenging for the human eye to detect, potentially leading to improved diagnostic performance. Furthermore, a noticeable trend has emerged within this subtopic, which is to combine other mechanisms with LLMs. For example, Wang et al [130] combined computer-aided diagnosis network with LLMs, which have shown amazing ability in reading chest X-rays. Similarly, Selivanov et al [127] combined a preset-attention mechanism with LLMs, which showcased efficient applicability to the chest X-ray image captioning task. This combination trend could be a promising future direction of LLMs development in medical image understanding.

#### Medical Question Answering

On the basis of our results, it is clear that diagnosis accuracy dominates a crucial part of research. This consists of enhancing diagnosis accuracy of LLMs and exploring the diagnosis accuracy of LLMs in various medical domains.

**Figure 5:**
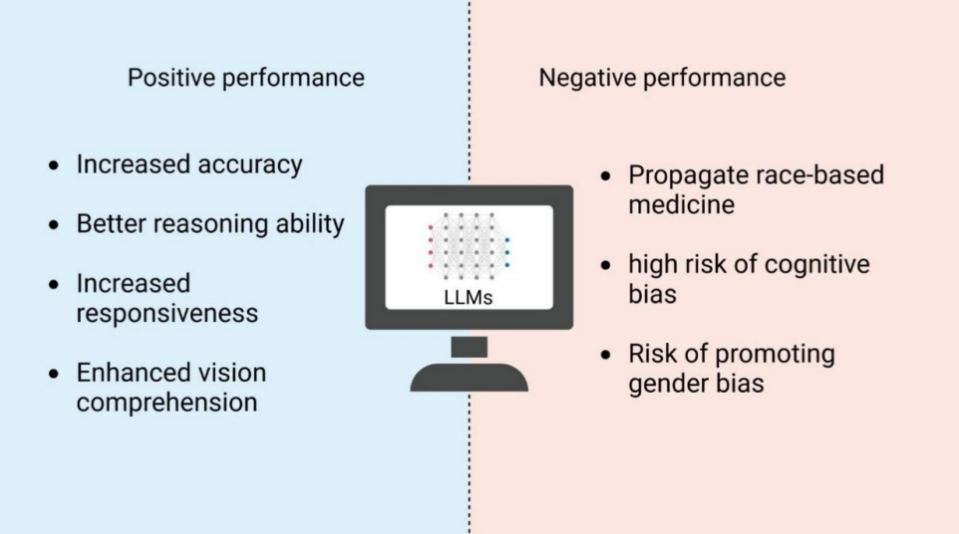
Positive and negative performance of current large language models (LLMs). Increased accuracy means LLMs have evolved to provide more accurate diagnosis results. Better reasoning ability suggests LLMs can analyze complex symptoms and make logical deductions. Increased responsiveness shows LLMs can respond to a wider range of queries. Enhanced vision comprehension suggests LLMs can interpret and understand visual data, which is beneficial for tasks like image analysis. Propagating race-based medicine suggests that there is a risk that these models could reinforce or propagate biased practices in medicine, leading to unequal treatment. High risk of cognitive bias reveals that the models might inherit and amplify biases present in the data they were given, affecting decision-making processes. The risk of promoting gender bias is similar to racial bias, which could also be perpetuated, affecting how information is processed and decisions are made.

One major indicator of diagnosis accuracy is medical-related questions and answers (QAs). Here, we listed the QA accuracy of LLM performance in Figure 4 and Multimedia Appendix 3 [20,131-138]. Models such as MedPaLM [20], GPT-4, and MedPaLM2 clearly indicate a trend that suggests a substantial increase in the accuracy of LLMs in answering questions in the MedQA dataset in a short period. In addition to the increased accuracy, a noticeable trend in developing medical task–specific LLMs for QAs was observed when combining the results of Figures 4 and 5. As previously discovered, the application of LLMs is constrained by their limited medical domain knowledge and the complexities of clinical tasks. For example, the performance of ChatGPT with human respondents in answering genetic questions was not significantly different from human respondents [139]. Given such observed limitations in medical knowledge, Li et al [140] introduced ChatDoctor, using the Llama model with an autonomous information retrieval mechanism. This allows real-time access and use of Wikipedia web-based resources, leading to a substantial enhancement in the quality of patient-physician interactive dialogue. The system has demonstrated notable progress in comprehending patient needs and offering precise treatment options. Yasunaga et al [141] developed DRAGON, a deep bidirectional language-knowledge graph pretraining method that enhances the model’s reasoning ability by providing knowledge graph that complements text data, offering structured background knowledge. Therefore, DRAGON achieved a higher score in QA across general and biomedical domains.

However, as most LLMs are trained on English corpora, advanced LLMs often struggle to perform effectively in non–English language settings, such as Chinese medical question answering systems. To address this limitation, researchers have focused on developing and applying Chinese-specific LLMs and datasets. For instance, Xiong et al [142] created DoctorGLM, a large-scale language model trained on a comprehensive Chinese health care database. DoctorGLM features a prompt designer module that extracts relevant keywords from user input, uses potential disease names as labels, and generates detailed descriptions based on a disease knowledge library. Therefore, DoctorGLM can provide users with accurate and reliable information, including disease symptoms, diagnoses, treatment options, and preventive measures. In addition, Li et al [21] introduced AcupunctureGPT, a model that not only supports the Chinese language but also integrates data from traditional Chinese medicine (TCM). By combining TCM principles with modern technology, AcupunctureGPT enhances diagnostic accuracy through objective methods and offers valuable scientific insights into the efficacy of TCM practices. This fusion of traditional knowledge and advanced AI represents a significant step forward in bridging the gap between ancient medical practices and contemporary health care technologies.

#### Quality of Generated Diagnostic Content

One major concern hindering LLMs’ use in medical diagnosis is closely related to the technical and ethical concerns that emerged in the diagnoses content.

Different from laboratory questions or QAs that give a clear indication and connection between symptoms and diseases, real-world clinical scenarios are often complex and sometimes misleading [143]. Therefore, it not only requires LLMs to have a deep understanding of medical knowledge but also the ability to handle ambiguity and uncertainty. For example, patients may present with atypical symptoms or nonspecific concerns that do not directly point to a particular disease. LLMs need to be capable of considering a broad differential diagnosis. It has been found that LLMs produce less accurate responses when faced with clinically biased questions as compared to unbiased ones, suggesting their vulnerability in dealing with the complexity of real patient-physician interactions [15].

More importantly, ethical problems shown in the diagnosis content of LLMs have caused more concerns in the field. This includes possible bias and privacy leakage problems during the diagnosing process. The importance of fairness in LLMs has become increasingly recognized for ensuring stable performance and unbiased diagnoses decisions. Language models, including LLMs, are known to reflect and sometimes amplify biases present in the historical data they learn from, which can exacerbate existing inequalities in health care [19]. For example, Omiye et al [144] revealed that every LLM model has instances of promoting race-based medicine or racist tropes or repeating unsubstantiated claims around race. Similarly, Yang et al [145] showed race-based tendencies in both GPT-3.5-turbo and GPT-4 models, including generating biased patient backgrounds, associating diseases with specific demographic groups, favoring White patients in treatment recommendations, and showing disparities in projected cost, hospitalization duration, and prognosis [145]. Such biased models could negatively impact the quality of diagnoses that patients receive. In addition, the diverse data sources used to train LLMs, including biomedical and clinical texts, may contain sensitive personal information, posing serious privacy risks [146]. Research has shown that language models can inadvertently leak personal information in their generated content [147]. Therefore, it is crucial to develop and deploy LLMs in medical diagnosis with robust mechanisms to prevent bias and protect patient privacy to ensure equitable and ethical health care outcomes.

### Quantitative Analysis of LLMs in Diagnostic Fields

The applications of LLMs in medical diagnosis varied and showed promise across several medical specialties. The frequency of LLM applications in different medical domains, as depicted in Table 5, indicates a high interest and potential in using these models for diagnostic purposes.

**Table 5:** Large language models’ use by diagnostic domains.

Diagnostic domains	Frequency, n
Radiology	16
General medicine	14
Neurology	10
Ophthalmology	10
Psychiatry	7
Emergency medicine	5
Cardiology	4
Dermatology	4
Dentistry	4
Surgery	3
Others	17

Among all the medical domains, research has focused on radiology, which accounts for 16 (17%) studies. In radiology, the application of LLMs is mainly focused on image recognition, interpretation, and diagnosis. LLMs can effectively identify pathological areas and improve diagnostic accuracy [148]. At the same time, LLMs can also conduct risk prediction and formulate personalized treatment plans based on patients’ medical imaging data [149]. In addition, through natural language generation technology, LLMs can also provide patients with understandable diagnostic reports and health guidance, which would aid the diagnostic process [150].

It is also worth noting that the application of LLMs on psychiatry diagnosis, including depression and attention-deficit/hyperactivity disorder are very promising [151-153]. Similarly, diseases of the nervous system also attract considerable attention, with studies covering disorders from predicting seizure to determining Alzheimer disease [154-156]. This might be because decision-making in psychiatry and neurology is particularly unique, as they have fewer objective tools that can be used to either confirm or refute a diagnosis [156]. Therefore, the emergence of LLMs can be an objective assessment tool that can help experts in decision-making process for diagnoses [121].

### Clinical Statistical Analysis

Clinical trials related to LLMs were searched in ClinicalTrials.gov (March 17, 2025) as indicators for LLMs’ real-world integration (Tables 6-8). After removing unrelated trials, 23 clinical trials were selected (Multimedia Appendix 4 provides detailed information on trials). These trials spanned across 11 medical specialties, with oncology (6/23, 26%) and ophthalmology (5/23, 22%) being the most extensively studied. All trials were funded by public sponsors and conducted in publicly available hospitals. None of the trials had reported their results by the date of search. Most trials (15/23, 65%) used LLMs as support in physicians whole diagnostic process, while some (6/23, 26%) investigated LLMs’ potential in direct diagnosis of diseases conducted by a physician. We observed that LLMs used for direct diagnosis were centered in emergency medicine and radiology. These areas showed shortages in physicians, where LLMs served as aid to reduce the workload of physicians [157,158]. For example, LLMs’ quick reasoning and responding makes them an ideal choice for grading emergency patients, and their enhanced vision understanding capabilities are valuable for generating radiology reports. It was found that ChatGPT and its variants still dominated the use in clinical trials (10/23, 43%), which is consistent with our previous findings. However, we observed a rise in using specifically develop LLMs in clinical settings compared with our previous findings (6/23, 26% vs. 9/95, 10%); this might be because the clinical use of LLMs was specialized in particular subject. Therefore, applying fine-tuning or prompt engineering method could enhance the usability of LLMs in expertized clinical scenario. In addition, it should be noted that certain trials did not report the LLMs used in the study or reported them without proper disclosure of their type (eg, only mentioned ChatGPT without its model types). Privacy concerns have driven innovations, such as locally deployed LLMs (ie, the ongoing trial registered as NCT06865534). These models minimize data transmission risks by processing information on-site, aligning with regulations like General Data Protection Regulation (GDPR). However, only this trial explicitly mentioned privacy safeguards, indicating a need for standardized reporting on data security measures in LLM research.

**Table 6:** Quantitative analysis of large language models (LLMs) used in clinical trials.

LLMs	Frequency (n=32), n (%)
ChatGPT	11 (34)
Gemini Pro	1 (3)
Marvin	2 (6)
Deepseek-Janus-Pro	1 (3)
Claude	4 (12)
Specifically developed LLM	6 (19)
Not reported	7 (22)

**Table 7:** The function of large language models (LLMs) in clinical trials. Note: the reason for having an intervention group is because one trial uses LLM both for diagnosis and intervention.

LLMs’ function	Frequency (n=23), n (%)
Diagnosis support	16 (70)
Direct diagnosis	6 (26)
Model comparison	1 (4)

**Table 8:** Large language models’ use in clinical trials by diagnostic domains.

Diagnostic domains	Frequency, n
Oncology	6
Ophthalmology	5
Cardiology	4
Pulmonology	3
General medicine	3
Gastroenterology	2
Neurology	2
Emergency medicine	2
Rheumatology	1
Radiology	1
Psychiatry	1

## Discussion

### Principal Findings

The field of LLMs in diagnosis has witnessed tremendous development in recent years, which is evident from our findings on study distribution across years. The results of our study contribute to a growing body of literature on the application of LLMs in health care. Previous scoping reviews have demonstrated that considerable research in the field of LLMs in health care is related to medical diagnosis [10]. In addition, there are existing literatures examining the current application of LLMs in diagnosis [159,160]. However, there is still a noticeable absence of a comprehensive analysis of LLMs in diagnosis to identify the gap between research and mapping the current research landscape of LLMs in medical diagnosis.

This is a pioneering study to quantitatively and comprehensively chart the integration of LLMs into the medical diagnosis domain. In this scoping review, we noticed a surge in publications regarding LLMs in diagnosis, signifying growth potential in this research field. This surge in research activity underscores the growing interest and potential impact of LLMs in transforming diagnostic processes in health care.

It is also worth noting that we performed a bibliometric analysis before our data extraction process. This was partly inspired by the bibliometric-systematic literature review approach proposed by Marzi et al [161]. In short, instead of gathering data directly from the 95 included articles, we performed a bibliometric analysis to cluster their keywords as indicators for determining research questions and data to extract. By using this technique, we were able to extract our needed information from a vast amount of data to support our findings. In addition, by performing a nested bibliometric analysis, this study was able to map the current landscape of LLM diagnostic performance evaluation, which provides valuable insight and is compatible with existing literature recommending a framework for future LLM performance evaluation [162].

Our review reveals that LLMs, particularly ChatGPT and its variants, show substantial promise in enhancing diagnostic accuracy and facilitating clinical decision-making. Traditional clinical decision support systems using decision trees increase users’ accuracy in diagnosing diseases [163] but struggle with complex presentations requiring probabilistic reasoning [164]. In contrast, LLMs demonstrate a superior diagnostic accuracy rate with a clearer indication of reasoning. However, the hallucination problem in LLMs creates regulatory challenges for high-stakes applications [165]. Nevertheless, the ability of LLMs to process vast amounts of textual and visual data enables them to classify diseases effectively, as demonstrated in various medical specialties such as radiology, psychiatry, neurology, and dermatology. For instance, GPT-4 has been successfully applied to detect and classify diseases from EHRs and medical images. Currently, the integration of LLMs with other technologies, such as computer-aided diagnosis networks and attention mechanisms, creates hybrid systems that combine the strengths of both approaches [130], which further enhances their diagnostic capabilities and suggests a promising direction for LLM development.

The rapid advancement in the accuracy of LLMs in medical QA tasks is another notable achievement. Models like MedPaLM, GPT-4, and MedPaLM2 have shown significant improvements in their ability to provide accurate and relevant answers to medical queries. This progress is partly attributed to the development of medical task–specific LLMs, which are fine-tuned on specialized datasets to enhance their domain knowledge and reasoning abilities. For example, ChatDoctor and AcupunctureGPT, which incorporate real-time information retrieval and traditional Chinese medicine principles, respectively, demonstrate the potential of LLMs to adapt to diverse medical contexts and improve diagnostic outcomes.

### Challenges Ahead

Despite promising advancements, several critical challenges remain. One of the most significant concerns is the potential for bias in LLMs, which can lead to inequitable and unethical health care outcomes. Our review highlights that LLMs are susceptible to reflecting and amplifying biases present in their training data, resulting in biased diagnostic recommendations and disparities in patient care. For instance, studies have shown that LLMs can exhibit race-based biases in medical diagnosis and patient information processing. Specifically, LLMs have been found to generate biased patient backgrounds and associate diseases with specific racial or ethnic groups. For example, GPT-3.5-turbo has been shown to attribute unwarranted details to patients based on their race or ethnicity, such as associating Black male patients with a safari trip in South Africa, and varying its diagnoses for different racial and ethnic groups even under identical conditions (eg, diagnosing HIV in Black patients, tuberculosis in Asian patients, and cysts in White patients) [145]. In addition, LLMs may project higher costs and longer hospitalizations for certain racial groups. For example, GPT-3.5-turbo predicts higher costs and longer hospital stays for White patients compared to other racial and ethnic groups, potentially reflecting real-world health care disparities. Furthermore, LLMs have been found to show overly optimistic outcomes in challenging medical scenarios with higher survival rates for some groups compared to others, which may negatively impact the quality of diagnoses and treatment decisions [144]. It should also be noted that apart from the race and gender bias that were directly reported by researchers studying medical diagnosis, LLMs had also exhibited bias in patient care regarding age, social status, and income level. These are also possible hidden bias that might affect the decision-making of LLMs in medical diagnosis.

Addressing these biases is crucial to ensure that LLMs provide fair and accurate diagnostic support to all patients. Currently, the most widely adopted method for bias mitigation is through prompt tuning. By carefully designing and adjusting the prompts used to query the LLMs, it is possible to guide the models toward more equitable and accurate responses. For example, researchers have found that using prompts that explicitly emphasize fairness and inclusivity can reduce the likelihood of biased outputs [166]. In addition, incorporating counternarratives or examples that challenge stereotypes within the prompts can help counteract the biases that may be present in the training data [167]. However, such methods only provide a shield to prevent the occurrence of bias in diagnosis without changing its core. Reinforcement learning from human feedback is another strategy that allows humans to grade the model’s responses, which helps correct some model outputs, particularly on sensitive questions with known online misinformation. However, such a method costs additional computing and could result in a more ambiguous answer in diagnosis. For example, the racial bias in cost prediction for GPT-4 has been reduced from 18% in GPT-3.5 to 5%, but the “uncertainty rate” increased from 16.25% to 29.46% [145]. Therefore, future research in finding a suitable way fully eradicate biased medical diagnosis, and constant monitoring of LLMs bias performance are important for the application of LLM in medical diagnosis.

In addition, the complexity of real-world clinical scenarios presents a challenge for LLMs. From a technical perspective, the first hurdle for LLMs deployment in real-world clinical settings is the ambiguity and uncertainty in unstructured inputs [15]. Unlike structured QA tasks, patient concerns (eg, “chest pain”) may correspond to dozens of potential diseases. LLMs must generate reasonable differential diagnoses despite lacking definitive information. Additional hurdles include the need for dynamic interaction and real-time feedback. LLMs in clinical consultations require interactive information gathering in follow-up history and test results, among others. Current LLMs’ limited context windows (eg, GPT-4’s 32,000 tokens) may lead to critical information loss. In addition, dynamically synthesized laboratory results, imaging reports, and other multimodal data are required for an accurate and reasonable diagnosis, but cross-modal alignment techniques remain underdeveloped in LLMs. Therefore, ensuring that LLMs can effectively navigate these complexities without compromising diagnostic accuracy is a critical area for future research.

Beyond technical challenges, the clinical application of LLMs faces multifaceted nontechnological barriers, including fragmented regulatory frameworks, physician skepticism, data privacy risks, ethical dilemmas, and regulatory inconsistencies across regions, such as the United States. The requirement of software as a medical device for consistent and reproducible results for medical software contradicts LLMs’ nature as products generating differentiated content for each answer. In addition, the United States’ requirement for predefined change control plans for adaptive algorithms, the European Union’s stringent transparency and data traceability mandates under the AI Act and the GDPR, and China’s life cycle quality control protocols create complex compliance landscapes that delay global deployment [168,169]. Clinician trust is further eroded by the black box nature of LLMs, concerns over data representativeness (eg, biases arising from existing studies), and ambiguous liability frameworks for errors [170]. Data privacy remains a critical hurdle, as LLMs rely on sensitive patient information while navigating strict anonymization rules (eg, the GDPR) and cross-border data flow restrictions. Current LLMs in medical diagnosis are still mainly commercially available models, with only a few reports on local deployment of LLMs, which raises the concern of patient data leakage. Ethical risks, such as algorithmic biases in gender or race and inadequate patient consent processes, compound public distrust, particularly among vulnerable populations [19]. These challenges underscore the need for international regulatory harmonization, enhanced algorithmic transparency through adversarial testing, and collaborative ethical governance to balance innovation with patient safety and trust in real-world clinical settings.

### Future Directions

#### Overview

Building on the current advancements and challenges identified in this review, we propose a structured research road map to guide the next phase of LLM development in medical diagnosis. In total, 4 critical domains warrant prioritized investigation.

#### Multimodal AI Integration for Holistic Diagnostics

Future studies should focus on integrating text, imaging, and structured clinical data (eg, laboratory results and genomic profiles) into unified multimodal frameworks. Although LLMs like GPT-4 have shown some success in processing EHRs and radiology reports, their ability to dynamically synthesize diverse data streams is still underdeveloped. Key priorities include developing cross-modal alignment techniques to harmonize semantic relationships between textual symptoms, imaging findings (eg, MRI anomalies), and biomarker patterns; creating benchmark datasets that reflect real-world clinical complexity, such as longitudinal patient records with asynchronous laboratory and imaging updates; and investigating hybrid architectures that combine LLMs with computer vision systems (eg, vision transformers) for simultaneous analysis of dermatology images and symptom narratives.

#### Enhancing Trust Through Explainable AI and Clinical Validation

To address physician skepticism and regulatory concerns, research must bridge the interpretability gap by developing chain-of-reasoning frameworks that visualize LLM diagnostic pathways, explicitly linking symptom inputs to disease hypotheses through intermediate evidence (eg, “Elevated CRP → infection likelihood → differential weighting of tuberculosis vs lymphoma”); implementing adversarial testing protocols to quantify model uncertainty in ambiguous presentations (eg, differentiating psychosomatic vs organic causes of chest pain); and conducting large-scale prospective trials comparing LLM-assisted versus conventional diagnostic workflows across institutions, with metrics including time-to-diagnosis, cost efficiency, and diagnostic error rates.

#### Specialty-Specific Optimization and Cross-Disciplinary Comparisons

The heterogeneous performance of LLMs across medical domains necessitates targeted investigations, including establishing specialty-specific evaluation benchmarks (eg, psychiatry: symptom trajectory modeling and oncology: rare cancer detection in pathology reports); conducting comparative studies analyzing performance variance across disciplines; and exploring transfer learning paradigms where diagnostic patterns learned in data-rich specialties (eg, radiology) inform models for underserved domains (eg, tropical medicine).

#### Ethical and Regulatory Harmonization

Parallel technical efforts must address systemic barriers to clinical implementation by developing international standards for bias auditing that require LLMs to demonstrate less demographic variance in diagnostic accuracy across protected attributes, such as race, gender, and socioeconomic status; creating federated learning infrastructures that enable secure multi-institutional training while complying with regulations like the GDPR and the Health Insurance Portability and Accountability Act through techniques such as differential privacy; and proposing adaptive regulatory frameworks that balance the probabilistic nature of LLM outputs with medical device safety requirements, potentially introducing real-time clinician oversight protocols for high-stakes diagnoses.

This road map emphasizes translational research that bridges technical innovation with clinical pragmatism. By focusing on multimodal integration, explainable reasoning, specialty-specific validation, and ethical governance, the field can transition from demonstrating diagnostic potential to delivering measurable improvements in patient outcomes. Collaborative efforts between AI researchers, clinicians, and policy makers will be essential to realize LLMs’ transformative potential while maintaining rigorous standards of care.

### Limitations of the Scoping Review Process

The scoping review process, while comprehensive, has inherent limitations. First, our reliance on selected databases might have excluded pertinent publications from nonindexed sources, for example, arXiv. Therefore, the source for this scoping review is limited. Despite that, we have tried to include articles from other sources by screening the citation of included studies; however, certain valuable gray literature or ongoing research may be omitted. In addition, this review lacked longitudinal studies evaluating the long-term clinical impact of LLMs, such as sustained diagnostic accuracy, clinician reliance, and patient outcomes over extended periods. This gap restricts conclusions about the durability of LLM performance and its integration into dynamic health care workflows. Moreover, because research into LLMs in medical diagnosis is only beginning, the rapid evolution of LLM technology introduces a temporal limitation: studies published after January 2025 or advancements in multimodal architectures may already outpace findings in this review. In addition, due to the expected heterogeneity in tasks and end points, we did not conduct formal meta-analyses or other statistical analysis. Instead, we presented simple descriptive statistics to provide an overview of the features of the LLMs’ landscape in medical diagnosis. Future updates with statistical analysis are critical to maintaining relevance in this fast-moving field.

### Conclusions

In conclusion, this scoping review highlights the significant potential of LLMs to revolutionize medical diagnostics, while also emphasizing the critical need to address biases, privacy concerns, and the complexities of real-world clinical scenarios. By focusing on these areas, future research can pave the way for the successful integration of LLMs into health care systems, ultimately improving diagnostic accuracy and patient outcomes.

## Appendix

Multimedia Appendix 1 Search strategy.

Multimedia Appendix 2 Characteristics of included studies.

Multimedia Appendix 3 Detailed data of large language models included in the accuracy results.

Multimedia Appendix 4 Characteristics of included clinical trials.

Multimedia Appendix 5 PRISMA-ScR checklist.

## Abbreviations

AI: artificial intelligence
EHR: electronic health record
GDPR: General Data Protection Regulation
LLM: large language model
PRISMA-ScR: Preferred Reporting Items for Systematic Reviews and Meta-Analyses extension for Scoping Reviews
QA: question and answer
TCM: traditional Chinese medicine

## Data Availability

The datasets used and analyzed during this study are available from the corresponding author on reasonable request.
