# Supplementary material for: Large Language Models in Medical Diagnostics: Scoping Review With Bibliometric Analysis
Source: J Med Internet Res. 2025 Jun 9;27:e72062. doi: 10.2196/72062 (PMC12186007; doi:10.2196/72062)
Supplement: Multimedia Appendix 1 [file jmir_v27i1e72062_app1.docx]

**Supplementary material 1.** Search strategy

1. Search strategies in Web of Science Core collection

Last searched January 2, 2025

| No. | Query | Results |
| --- | --- | --- |
| #1 | (((TS=(large language model)) OR TS=(chatbot*)) OR TS=(transformer*)) OR TS=(LLM) OR TS=(‘BERT’) OR TS=(‘GPT’) | 97,282 |
| #2 | (((((((TS=(diagnose)) OR TS=(diagnosi*)) OR TS=(diagnosed)) OR TS=(diagnosing)) OR TS=(diagnosable)) OR TS=(diagnostic)) OR TS=(diagnostics)OR TS=(diagnostician)) OR TS=(diagnoser) | 2,948,159 |
| #3 | #1 AND #2 AND and 2025 or 2024 or 2023 or 2022 (Publication Years) | 4,775 |

Note: TS=Topic (Searches title, abstract, author keywords, and Keywords Plus.)

2. Search strategies in Pubmed

| No | Search strategy | Results |
| --- | --- | --- |
| #1 | "diagnosable"[Title/Abstract] OR "diagnosi*"[Title/Abstract] OR "diagnosis"[MeSH Terms] OR "diagnose"[Title/Abstract] OR "diagnosed"[Title/Abstract] OR "diagnoses"[Title/Abstract] OR "diagnosing"[Title/Abstract] OR "diagnosis"[MeSH Subheading] OR "diagnostic"[Title/Abstract] OR "diagnostics"[Title/Abstract] OR "diagnoser"[Title/Abstract] OR "diagnostician"[Title/Abstract] | 12,224,269 |
| #2 | "LLM"[Title/Abstract] OR "Large language model"[Title/Abstract] OR "BERT"[Title/Abstract] OR "chatbot*"[Title/Abstract] OR "transformer"[Title/Abstract] OR “GPT*"[Title/Abstract] | 14,564 |
| #3 | #1 AND #2 AND "2022"[Date - Publication] : "2024"[Date - Publication] | 3,270 |

3. Search strategies in Embase

| No. | Query | Results |
| --- | --- | --- |
| #1 | 'diagnostic procedure':ab,ti OR 'diagnosis'/exp OR diagnosi*:ab,ti OR diagnose:ab,ti OR diagnosing:ab,ti OR diagnostic:ab,ti OR diagnostics:ab,ti OR diagnosed:ab,ti OR diagnostician:ab,ti OR diagnoser:ab,ti | 10,935,814 |
| #2 | 'large language model’/exp OR ‘large language model’:ab,ti OR ‘transformer*’:ab,ti OR ‘LLM’:ab,ti OR ‘chatbot*’:ab,ti OR ‘BERT’:ab,ti | 18,995 |
| #3 | #1 AND #2 AND [2022-2024]/py | 5,399 |

1. Search strategies in IEEE Xplore

| No. | Query | Results |
| --- | --- | --- |
| #1 | "All Metadata":large language model OR "All Metadata":chatbot OR "All Metadata":LLM OR "All Metadata":transformer OR "All Metadata":BERT | 190,400 |
| #2 | "All Metadata":diagnosis | 118,376 |
| #3 | #1 AND #2 Filters Applied: 2022 - 2024 | 3,054 |

1. Search strategies in ACM Digital Library

| No. | Query | Results |
| --- | --- | --- |
| #1 | [Abstract: 'large language model'] OR [Abstract: llm] OR [Abstract: bert] OR [Abstract: chatbot] OR [Abstract: transformer] | 190,400 |
| #2 | [All: diagnose] OR [All: diagnosis] | 47,053 |
| #3 | #1 AND #2 AND [E-Publication Date: (01/01/2022 TO 12/31/2024)] | 1,259 |
