# Supplementary material for: Large Language Models in Medical Diagnostics: Scoping Review With Bibliometric Analysis
Source: J Med Internet Res. 2025 Jun 9;27:e72062. doi: 10.2196/72062 (PMC12186007; doi:10.2196/72062)
Supplement: Multimedia Appendix 2 [file jmir_v27i1e72062_app2.docx]

**Supplementary material 2.** Characteristic of included studies

| **Title** | **Author** | **Specialty** | **Task** | **Primary endpoint** | **Comparison** | **Primary Result** | **LLM type** | **Article label** |
| --- | --- | --- | --- | --- | --- | --- | --- | --- |
| Taming large language models to implement diagnosis and evaluating the generation of LLMs at the semantic similarity level in acupuncture and moxibustion | Li, SS et al. | Traditional Chinese Medicine | Diagnosis and Treatment Planning | Semantic Similarity | AcupunctureGPT vs. Other LLMs | AcupunctureGPT outperforms other LLMs in diagnosing diseases and devising treatment plans | AcupunctureGPT (Prompt engineering) | Cluster 1 & Cluster 2 |
| Evaluating capabilities of large language models: Performance of GPT-4 on surgical knowledge assessments | Beaulieu-Jones, BR et al. | Surgery | Knowledge Assessment | Diagnostic Accuracy | GPT-4 vs. Surgical knowledge assessments | GPT-4: 71.3% accuracy on multiple-choice, 47.9% on open-ended questions | GPT-4 | Cluster 1 & Cluster 2 |
| The Diagnostic Ability of GPT-3.5 and GPT-4.0 in Surgery: Comparative Analysis | Liu, JY et al. | Surgery | Diagnostic Accuracy | Primary and Secondary Diagnoses | GPT-3.5 vs. GPT-4.0 | GPT-4.0 accuracy higher than GPT-3.5 (Primary: 97.2% vs 85.5%; Secondary: 90.8% vs 61.7%) | GPT-3.5, GPT-4.0 | Cluster 1 & Cluster 2 |
| AI Versus MD: Evaluating the surgical decision-making accuracy of ChatGPT-4 | Palenzuela, Deanna L. et al. | Surgery | Surgical Decision-Making | Diagnostic Accuracy | ChatGPT-4 vs. Surgeons | ChatGPT-4 superior to junior residents | ChatGPT-4 | Cluster 1 & Cluster 2 |
| Data augmentation based on large language models for radiological report classification | Collado-Montanez, Jaime et al. | Radiology | Report Classification | Classification Performance | - | Enhanced classification performance | RoBERTa biomedical–clinical model + fine tuning | Cluster 1 & Cluster 2 |
| ChatGPT's diagnostic performance based on textual vs. visual information compared to radiologists' diagnostic performance in musculoskeletal radiology | Horiuchi, D et al. | Radiology | Diagnostic Accuracy | Accuracy rates of GPT-4-based ChatGPT, GPT-4V-based ChatGPT, and radiologists | GPT-4-based ChatGPT vs. GPT-4V-based ChatGPT vs. Radiologists | GPT-4-based ChatGPT: 43% accuracy (46/106); GPT-4V-based ChatGPT: 8% accuracy (9/106); Radiologists: 41% (resident) and 53% (board-certified) | GPT-4, GPT-4V | Cluster 1 & Cluster 2 |
| Feasibility of large language models for CEUS LI-RADS categorization of small liver nodules in patients at risk for hepatocellular carcinoma | Huang, JY et al. | Radiology | Disease classification | Sensitivity and specificity | ChatGPT-4.0 vs. ChatGPT-4o | ChatGPT-4.0 superior in sensitivity | ChatGPT-4.0, ChatGPT-4o | Cluster 1 & Cluster 2 |
| Revolution or risk?-Assessing the potential and challenges of GPT-4V in radiologic image interpretation | Huppertz, MS et al. | Radiology | Image interpretation | Diagnostic accuracy | GPT-4V vs. Radiologists | GPT-4V accuracy 8.3% (uncontextualized), 29.1% (contextualized) | GPT-4V | Cluster 1 & Cluster 2 |
| Toward Improved Radiologic Diagnostics: Investigating the Utility and Limitations of GPT-3.5 Turbo and GPT-4 with Quiz Cases | Kikuchi, T et al. | Radiology | Diagnostic accuracy | Correct response rate | GPT-3.5 Turbo vs. GPT-4 | GPT-3.5 Turbo 26%, GPT-4 41% | GPT-3.5 Turbo, GPT-4 | Cluster 1 |
| Harnessing Large Language Models for Structured Reporting in Breast Ultrasound: A Comparative Study of Open AI (GPT-4.0) and Microsoft Bing (GPT-4) | Liu, CX et al. | Radiology | Structured reporting | Structured report quality | OpenAI GPT-4.0 vs. Microsoft Bing GPT-4 | OpenAI GPT-4.0 better in structured reports (88% vs. 55%) | GPT-4.0, Bing GPT-4 | Cluster 1 & Cluster 2 |
| Comparative analysis of GPT-4-based ChatGPT's diagnostic performance with radiologists using real-world radiology reports of brain tumors | Mitsuyama, Y | Radiology | Diagnostic Accuracy | Final and Differential Diagnoses | GPT-4 vs. Radiologists | GPT-4 final diagnostic accuracy: 73%; Differential accuracy: 94% | GPT-4 | Cluster 1 & Cluster 2 |
| Large language model may assist diagnosis of SAPHO syndrome by bone scintigraphy | Mori, Y et al. | Radiology | Diagnosis of SAPHO syndrome | Sensitivity, Specificity, Accuracy | LLM vs. Rheumatologists | Sensitivity 83.5%, Specificity 69.4%, Accuracy 76.8% | ChatGPT-4 | Cluster 1 & Cluster 2 |
| Preliminary assessment of automated radiology report generation with generative pre-trained transformers: comparing results to radiologist-generated reports | Nakaura, T et al. | Radiology | Report accuracy | Top-1, Top-5 accuracy | GPT-2, GPT-3.5, GPT-4 vs. Radiologists | Radiologists best, GPT-4 highest among LLMs | GPT-2, GPT-3.5, GPT-4 | Cluster 1 & Cluster 2 |
| Capability of multimodal large language models to interpret pediatric radiological images | Reith, TP et al. | Radiology | Image Interpretation | Diagnostic Accuracy | GPT-4, Gemini 1.5 Pro, Claude 3 Opus vs. Human Experts | Overall correct diagnosis rate: 27.8% (25/90); Partially correct: 13.3% (12/90); Incorrect: 58.9% (53/90) | GPT-4, Gemini 1.5 Pro,Claude 3 Opus | Cluster 1 & Cluster 2 |
| Performance of a commercially available Generative Pre-trained Transformer (GPT) in describing radiolucent lesions in panoramic radiographs and establishing differential diagnoses | Silva, TP et al. | Radiology | Descriptive and Differential Diagnoses | Description accuracy, Differential diagnosis classification | GPT-3.5 vs. Examiners | Description accuracy: Margination 0.93, Affected bone 0.93; Differential diagnosis accuracy: Rank-1 25%, Rank-3 67.85% | GPT-3.5 | Cluster 1 & Cluster 2 |
| PneumoLLM: Harnessing the power of large language model for pneumoconiosis diagnosis | Song, MY et al. | Radiology | Disease Diagnosis | Accuracy in diagnosing pneumoconiosis | Adapter layers vs. Classification head | Improved diagnostic accuracy with fewer learnable parameters | PneumoLLM | Cluster 1 & Cluster 2 |
| Diagnostic performances of GPT-4o, Claude 3 Opus, and Gemini 1.5 Pro in "Diagnosis Please" cases | Sonoda, Y et al. | Radiology | Diagnostic accuracy | Primary diagnosis accuracy | GPT-4o, Claude 3 Opus, Gemini 1.5 Pro | Claude 3 Opus 54%, GPT-4o 41%, Gemini 1.5 Pro 34% | GPT-4o, Claude 3 Opus, Gemini 1.5 Pro | Cluster 1 & Cluster 2 |
| Comparing Diagnostic Accuracy of Radiologists versus GPT-4V and Gemini Pro Vision Using Image Inputs from Diagnosis Please Cases | Suh, PS et al. | Radiology | Diagnostic accuracy | Correct diagnosis | GPT-4V, Gemini Pro Vision vs. Radiologists | GPT-4V accuracy 49%, Radiologists 61% | GPT-4V, Gemini Pro Vision | Cluster 1 & Cluster 2 |
| Testing the Ability and Limitations of ChatGPT to Generate Differential Diagnoses from Transcribed Radiologic Findings | Sun, SH et al. | Radiology | Differential Diagnoses | Accuracy, Reliability, Repeatability | GPT-3.5 vs. GPT-4 | GPT-4: Final diagnosis accuracy 66.1%, Differential score 0.54; GPT-3.5: Final diagnosis accuracy 53.7%, Differential score 0.50 | GPT-3.5/GPT-4 | Cluster 1 & Cluster 2 |
| Performance of Progressive Generations of GPT on an Exam Designed for Certifying Physicians as Certified Clinical Densitometrists | Valdez, D et al. | Radiology | Diagnostic accuracy | Score and accuracy | GPT-3 vs. GPT-4 | GPT-4 passing score 342, GPT-3 failing score 289 | GPT-3, GPT-4 | Cluster 1 & Cluster 2 |
| Mental Health Prediction from Social Media Text Using Mixture of Experts | dos Santos, WR et al. | Psychiatry | Mental Health Prediction | Accuracy in predicting depression/anxiety disorders | Conventional engineered features vs. Transformer-based models | GPT-4-based models showed higher accuracy in predicting mental health conditions | GPT-4 | Cluster 1 & Cluster 2 |
| Appraising the performance of ChatGPT in psychiatry using 100 clinical case vignettes | D'Souza, RF et al. | Psychiatry | Diagnostic accuracy | ChatGPT performance in psychiatry | Clinical case vignettes | High performance in management strategies | ChatGPT 3.5 | Cluster 1 & Cluster 2 |
| Diagnostic accuracy of large language models in psychiatry | Gargari, OK et al. | Psychiatry | Diagnostic accuracy | Accuracy and reasoning quality | GPT-3.5, GPT-4, Aya, Nemotron | GPT-3.5 and GPT-4 superior in accuracy and reasoning | GPT-3.5, GPT-4 | Cluster 1 & Cluster 2 |
| Large language models outperform mental and medical health care professionals in identifying obsessive-compulsive disorder | Kim, J et al. | Psychiatry | Diagnostic accuracy | Correct diagnosis | LLMs vs. Mental health professionals | LLMs outperform professionals in OCD diagnosis | ChatGPT-4, Gemini Pro, and Llama 3 | Cluster 1 & Cluster 2 |
| Large language models outperform general practitioners in identifying complex cases of childhood anxiety | Levkovich, I et al. | Psychiatry | Diagnosis of childhood anxiety | Diagnostic accuracy | LLMs vs. GPs | LLMs significantly better than GPs | ChatGPT-3.5 and ChatGPT-4, Claude.AI, Gemini | Cluster 1 & Cluster 2 |
| A Hybrid System Based on Bayesian Networks and Deep Learning for Explainable Mental Health Diagnosis | Pavez, J et al. | Psychiatry | Mental health diagnosis | Symptom checker tool accuracy | Large Language model + Bayesian Networks | High accuracy in classifying mental health disorders | BERTbase | Cluster 1 & Cluster 2 |
| Using Large Language Models to Detect Depression From User-Generated Diary Text Data as a Novel Approach in Digital Mental Health Screening: Instrument Validation Study | Shin, D et al. | Psychiatry | Depression detection | Detection accuracy | GPT-3.5 vs. GPT-4 | GPT-3.5 fine-tuning shows superior performance in depression detection | GPT-3.5, GPT-4 | Cluster 1 & Cluster 2 |
| Performance of ChatGPT Compared to Clinical Practice Guidelines in Making Informed Decisions for Lumbosacral Radicular Pain: A Cross-sectional Study | Gianola, S et al. | Physiatry | Lumbosacral radicular pain management | Accuracy vs. CPGs | ChatGPT vs. Clinical Practice Guidelines | ChatGPT accuracy 33% | GPT-3.5 | Cluster 1 & Cluster 2 & Cluster 3 |
| Diagnostic Accuracy of a Custom Large Language Model on Rare Pediatric Disease Case Reports | Young, CC et al. | Pediatrics | Diagnostic accuracy | Diagnostic accuracy | GPT-4, Gemini Pro, Custom LLM (GPT-4 HPO) | GPT-4 diagnostic accuracy 13.1%, Custom LLM 8.2% | GPT-4, GPT-4HPO | Cluster 1 & Cluster 2 |
| Comparing the Quality of Domain-Specific Versus General Language Models for Artificial Intelligence-Generated Differential Diagnoses in PICU Patients | Akhondi-Asl, A et al. | Pediatric Critical Care | Differential Diagnoses | Quality of diagnoses | Fine-tuned LLMs vs. General LLMs | Fine-tuned LLaMa-7B outperformed general LLMs | Fine-tuned LLaMa-7B | Cluster 1 & Cluster 2 |
| Comparing customized ChatGPT and pathology residents in histopathologic description and diagnosis of common diseases | Apornvirat, Sompon et al. | Pathology | Histopathologic Diagnosis | Diagnostic Accuracy | ChatGPT vs. Residents | Residents outperformed ChatGPT | GPT-4 | Cluster 1 & Cluster 2 |
| Exploring the role of ChatGPT in clinical decision-making in otorhinolaryngology: a ChatGPT designed study | Teixeira-Marques, F et al. | Otorhinolaryngology | Clinical decision-making | Agreement with ENT specialists | ChatGPT vs. ENT specialists | ChatGPT mean score 4.4 | ChatGPT-1，ChatGPT-2 | Cluster 1 & Cluster 2 |
| Application of ChatGPT as a support tool in the diagnosis and management of acute bacterial tonsillitis | Miguel et al. | Otorhinolaryngology | Diagnosis and Treatment Planning | Diagnostic Accuracy; Recommendation quality | - | GPT-3.5 achieved accurate diagnoses in 100% of cases | GPT-3.5 | Cluster 1 & Cluster 2 |
| ChatGPT-4 Consistency in Interpreting Laryngeal Clinical Images of Common Lesions and Disorders | Maniaci, A et al. | Otolaryngology | Image interpretation | Consistency in image analysis | ChatGPT-4 vs. Laryngologists | ChatGPT-4 primary diagnosis accuracy 20.0% to 25.0% | ChatGPT-4 | Cluster 1 & Cluster 2 |
| Quantitative evaluation of GPT-4's performance on US and Chinese osteoarthritis treatment guideline interpretation and orthopaedic case consultation | Li, JT et al. | Orthopedics | Guideline Interpretation and Case Consultation | Accuracy and completeness of responses | GPT-4 vs. Orthopedic specialists | High accuracy (88%) in case-based questions | GPT-4 | Cluster 1 & Cluster 3 |
| Arthrosis diagnosis and treatment recommendations in clinical practice: an exploratory investigation with the generative AI model GPT-4 | Pagano, S et al. | Orthopedics | Diagnosis and Treatment Recommendations | Agreement with clinical decisions | GPT-4 vs. Orthopedic specialists | 83% agreement in treatment recommendations; Sensitivity: 78%, Specificity: 80% for surgical treatment | GPT-4 | Cluster 1 & Cluster 2 & Cluster 3 |
| Large Language Models for Therapy Recommendations Across 3 Clinical Specialties: Comparative Study | Wilhelm, TI et al. | Ophthalmology, Orthopedics, Dermatology | Therapy Recommendations | Correctness and Safety of Recommendations | Claude-instant-v1.0, GPT-3.5-Turbo, Command-xlarge-nightly, Bloomz | Claude-instant-v1.0 highest mDISCERN score (3.35); Bloomz lowest score (1.07) | GPT-3.5-Turbo, Command-xlarge-nightly, Claude-instant-v1.0,Bloomz | Cluster 1 & Cluster 2 & Cluster 3 |
| ICGA-GPT: report generation and question answering for indocyanine green angiography images | Chen, XL et al. | Ophthalmology | Report accuracy and QA | Report quality, Diagnostic Accuracy | GPT-4 vs. Human | GPT-4: High report quality (BLEU 0.48, CIDEr 0.82); Accuracy metrics: Specificity 0.98, Accuracy 0.94 | GPT-4 | Cluster 1 & Cluster 2 & Cluster 3 |
| Performance of ChatGPT in Diagnosis of Corneal Eye Diseases | Delsoz, M et al. | Ophthalmology | Corneal disease diagnosis | Diagnostic accuracy | ChatGPT-4.0 vs. ChatGPT-3.5 vs. Human experts | ChatGPT-4.0 accuracy 85% | ChatGPT-4.0, ChatGPT-3.5 | Cluster 1 & Cluster 2 |
| What can GPT-4 do for Diagnosing Rare Eye Diseases? A Pilot Study | Hu, XY et al. | Ophthalmology | Diagnostic accuracy | Suitability and accuracy | GPT-4 vs. Human experts | GPT-4 83.3% appropriate, 90% accurate in junior ophthalmologist scenario | GPT-4 | Cluster 1 & Cluster 2 |
| Performance of ChatGPT in French language analysis of multimodal retinal cases | Mikhail, D et al. | Ophthalmology | Multimodal diagnosis | Diagnostic accuracy | ChatGPT-4 vs. Human experts | ChatGPT-4 accuracy 35.8% (English), 28.4% (French) | ChatGPT-4 | Cluster 1 & Cluster 2 |
| Performance of ChatGPT in Ophthalmic Registration and Clinical Diagnosis: Cross-Sectional Study | Ming, S et al. | Ophthalmology | Registration and Diagnosis | Diagnostic Accuracy | GPT-3.5 vs. GPT-4 | GPT-4: 77.9% registration accuracy, 60.6% diagnosis accuracy; GPT-3.5: 63.5% registration accuracy, 39.4% diagnosis accuracy | GPT-3.5/GPT-4 | Cluster 1 & Cluster 2 |
| Chatbots Vs. Human Experts: Evaluating Diagnostic Performance of Chatbots in Uveitis and the Perspectives on AI Adoption in Ophthalmology | Rojas-Carabali, W et al. | Ophthalmology | Uveitis diagnosis | Diagnostic accuracy | ChatGPT vs. Uveitis experts | ChatGPT 66% success rate | GPT-3.5, GPT-4，Glass 1.0 | Cluster 1 & Cluster 2 |
| Unveiling the clinical incapabilities: a benchmarking study of GPT-4V(ision) for ophthalmic multimodal image analysis | Xu, PS et al. | Ophthalmology | Multimodal Image Analysis | Diagnostic accuracy | GPT-4V-based ChatGPT vs. Human readers | GPT-4V: 30.6% accurate, 21.5% highly usable, 55.6% no harm; Sensitivity: 24.0%, Specificity: 25.6% | GPT-4V | Cluster 1 & Cluster 2 |
| Understanding natural language: Potential application of large language models to ophthalmology | Yang, ZF et al. | Ophthalmology | Ophthalmology classification | Diagnostic accuracy | Various LLMs | High accuracy in classifying genetic conditions | Bing AI, Docs-GPT Beta，ChatGPT-3.5，Bard，GPT-4，ChatFFA，FFA-GPT，ICGA-GPT，Google Bard，OphGLM，PaLM2，Bing Chat，Claude-instant-v1.0, | Cluster 1 & Cluster 2 |
| Exploring Diagnostic Precision and Triage Proficiency: A Comparative Study of GPT-4 and Bard in Addressing Common Ophthalmic Complaints | Zandi, R | Ophthalmology | Diagnostic Accuracy and Triage | Correct Leading Diagnosis | GPT-4 vs. Bard | GPT-4 better at triage (96.3%) and diagnosis (90.0% appropriate triage vs. 48.8% correct leading diagnosis) | GPT-4, Bard | Cluster 1 & Cluster 2 |
| A comparative study of GPT-4o and human ophthalmologists in glaucoma diagnosis | Zhang, JX et al. | Ophthalmology | Diagnostic accuracy | Primary and differential diagnosis accuracy | GPT-4o vs. Human ophthalmologists | GPT-4o lower in primary diagnosis, comparable in differential diagnosis | GPT-4o | Cluster 1 & Cluster 2 |
| The interaction of structured data using openEHR and large Language models for clinical decision support in prostate cancer | Kaiser, P et al. | Oncology | Clinical decision support | Adherence to treatment recommendations | LLMs vs. MDT | 93% adherence to MDT recommendations | ChatGPT-4, Claude-3-Opus | Cluster 1 & Cluster 2 & Cluster 3 |
| Chat-GPT on brain tumors: An examination of Artificial Intelligence/Machine Learning's ability to provide diagnoses and treatment plans for example neuro-oncology cases | Kozel, G et al. | Neurosurgery | Brain tumor diagnosis | Diagnostic accuracy | ChatGPT-3.5 vs. ChatGPT-4 | ChatGPT-4 accuracy 85% for diagnosis | ChatGPT-3.5, ChatGPT-4 | Cluster 1 & Cluster 2 |
| Can Artificial Intelligence Mitigate Missed Diagnoses by Generating Differential Diagnoses for Neurosurgeons? | Kumar, RP et al. | Neurosurgery | Differential Diagnosis | Accuracy in generating differential diagnoses | ChatGPT-3.5, ChatGPT-4, Perplexity AI, Bard AI | ChatGPT-4: 53.68% accuracy in initial differential diagnosis | ChatGPT-3.5, ChatGPT-4 | Cluster 1 & Cluster 2 |
| A Quantitative Assessment of ChatGPT as a Neurosurgical Triaging Tool | Ward, M et al. | Neurosurgery | Diagnostic Accuracy and Triage | Accuracy vs. Surgeons and Residents | GPT-3.5 vs. GPT-4 | GPT-4 100% accurate in triage and diagnosis; Superior to GPT-3.5 | GPT-3.5, GPT-4 | Cluster 1 & Cluster 2 |
| Generative pre-trained transformer (GPT)-4 support for differential diagnosis in neuroradiology | Sorin, V et al. | Neuroradiology | Differential diagnosis | Correct diagnosis | GPT-4 vs. Neuroradiologists | GPT-4 accuracy 61.7% (top-3 differential), Neuroradiologists 63.3%-73.3% | GPT-4 | Cluster 1 & Cluster 2 |
| Large language models as a diagnostic support tool in neuropathology | Hewitt, KJ et al. | Neuropathology | Diagnostic Accuracy | Accuracy in diagnosing CNS tumours | ChatGPT-4o, Claude-3.5-sonnet, Llama3 vs. WHO guidelines | LLMs with Retrieval-Augmented Generation (RAG) achieved 90% accuracy in diagnosing neuropathological tumour subtypes | ChatGPT-4o, Claude-3.5-sonnet, Llama3 | Cluster 1 & Cluster 2 |
| Customized GPT model largely increases surgery decision accuracy for pharmaco-resistant epilepsy | Chiang, KL et al. | Neurology | Surgery Decision Accuracy | Diagnostic Accuracy | Customized GPT Model vs. Protege-based Knowledge Base | Customized GPT Model accuracy: 93.8% | The JSON Epilepsy Matcher | Cluster 1 & Cluster 2 |
| Explainable cognitive decline detection in free dialogues with a Machine Learning approach based on pre-trained Large Language Models | de Arriba-Pérez, F et al. | Neurology | Cognitive Decline Detection | Detection accuracy in free dialogues | ChatGPT vs. Specialized Machine Learning models | ChatGPT achieved high performance in detecting cognitive decline | gpt-3.5-turbo8 | Cluster 1 & Cluster 2 |
| Evaluating the performance of large language models: ChatGPT and Google Bard in generating differential diagnoses in clinicopathological conferences of neurodegenerative disorders | Koga, S et al. | Neurology | Differential Diagnoses | Correct Diagnoses | ChatGPT-3.5, ChatGPT-4, Google Bard | ChatGPT-4 correct primary diagnoses: 52%; Google Bard: 40% | ChatGPT-3.5, ChatGPT-4, Google Bard | Cluster 1 & Cluster 2 |
| Artificial intelligence classifies primary progressive aphasia from connected speech | Rezaii, N et al. | Neurology | Speech Analysis | Classification accuracy of PPA variants | LLMs vs. Linguistic features | 97.9% accuracy in classifying PPA variants | GPT-4 | Cluster 1 & Cluster 2 |
| Text Dialogue Analysis for Primary Screening of Mild Cognitive Impairment: Development and Validation Study | Wang, CY et al. | Neurology | Validation Study | Screening for MCI using text dialogues | ChatGPT performance | High sensitivity and specificity | ChatGPT-3.5 and ChatGPT-4 | Cluster 1 & Cluster 2 |
| How to improve ChatGPT performance for nephrologists: a technique guide | Miao, J et al. | Nephrology | Performance Improvement | Diagnostic accuracy | Chain-of-thought vs. Standard prompting | Chain-of-thought method improved diagnostic accuracy | GPT-4 | Cluster 1 |
| Performance of ChatGPT on Chinese Master's Degree Entrance Examination in Clinical Medicine | Li, Ke-Cheng et al. | General Medicine | Examination Performance | Diagnostic Accuracy | ChatGPT-3.5 vs. GPT-4 | Both surpassed admission threshold | ChatGPT-3.5, GPT-4 | Cluster 1 |
| Integrating AI in Lipedema Management: Assessing the Efficacy of GPT-4 as a Consultation Assistant | Leypold, T et al. | Lipedema management | Consultation assistance | Reliability and applicability | GPT-4 vs. Human experts | GPT-4 average score 4.24 (Likert scale) | GPT-4 | Cluster 1 & Cluster 2 |
| Evaluation of large language models as a diagnostic aid for complex medical cases | Rios-Hoyo, A et al. | Internal Medicine | Diagnostic accuracy | Diagnostic Accuracy | GPT-3.5 vs. GPT-4 | GPT-4 better at listing correct diagnosis | GPT-3.5, GPT-4 | Cluster 1 |
| Exploring the potential of large language models in identifying metabolic dysfunction-associated steatotic liver disease: A comparative study of non-invasive tests and artificial intelligence-generated responses | Wu, WY et al. | Hepatology | Diagnostic accuracy | Diagnostic accuracy | GPT-3.5, GPT-4 vs. FLI, USFLI | GPT-4 AUROC 0.831, comparable to FLI and USFLI | GPT-3.5, GPT-4 | Cluster 1 |
| Assessing the potential of GPT-4 to perpetuate racial and gender biases in health care: a model evaluation study | Zack, T et al. | Healthcare | Bias assessment | Demographic representation | GPT-4 vs. True prevalence | GPT-4 shows significant biases in demographic representation | GPT-4 | Cluster 3 |
| On the role of the UMLS in supporting diagnosis generation proposed by Large Language Models | Afshar, Majid et al. | General Medicine | Diagnosis Generation | Diagnostic Accuracy | LLMs with UMLS | Improved diagnostic accuracy with UMLS | GPT-3.5 | Cluster 1 |
| Mixed methods assessment of the influence of demographics on medical advice of ChatGPT | Andreadis, K et al. | General Medicine | Diagnostic Accuracy and Health Advice | Demographic Tailoring | ChatGPT (GPT-4) vs. WebMD Symptom Checker | ChatGPT matches WebMD in 91% of diagnoses; No significant demographic bias | GPT-4 | Cluster 1 |
| Qualitative metrics from the biomedical literature for evaluating large language models in clinical decision-making | Ho, CN et al. | General Medicine | Diagnostic Accuracy, quality of diagnosed content | Accuracy, completeness, appropriateness | Various LLMs | GPT-4.0 highest in performance | GPT-4 | Cluster 1 |
| EHR-KnowGen: Knowledge-enhanced multimodal learning for disease diagnosis generation | Niu, S et al. | General Medicine | Disease Diagnosis | Accuracy in diagnosing diseases from EHR data | Multimodal learning with external domain knowledge | Superior performance over comparative methods in real-world EHR datasets | GPT-4 | Cluster 1 |
| Identifying symptom etiologies using syntactic patterns and large language models | Taub-Tabib, H et al. | General Medicine | Etiology identification | Precision, coverage | Syntactic patterns vs. GPT-4 | GPT-4 high precision, lower coverage | GPT-4 | Cluster 1 |
| The potential and pitfalls of using a large language model such as ChatGPT, GPT-4, or LLaMA as a clinical assistant | Zhang, JQ et al. | General Medicine | Diagnostic accuracy | F1-score | GPT-4 vs. Disease-specific ML models | GPT-4 F1-score >= 85% | GPT-4, LLaMA | Cluster 1 |
| Are Different Versions of ChatGPT’s Ability Comparable to the Clinical Diagnosis Presented in Case Reports? A Descriptive Study | Chen JF et al. | General Medicine | Diagnostic Accuracy | Primary and Secondary Diagnoses | GPT-4 vs. GPT-3.5 | GPT-4.0 scored 1.26, GPT-3.5 scored 1.01 | GPT-4.0, GPT-3.5 | Cluster 1 |
| Toward expert-level medical question answering with large language models | Karan Singhal et al. | General Medicine | Diagnostic accuracy | Score in MedQA, MedMCQA, PubMedQA, and MMLU clinical topics datasets | Med-PaLM 2 question answering | Med-PaLM 2 scores up to 86.5% on the MedQA dataset | Med-PaLM 2 (Fine tuning) | Cluster 1 |
| Domain-Specific Language Model Pretraining for Biomedical Natural Language Processing | Yu Gu et al. | General Medicine | Diagnostic accuracy | Score in PubMedQA dataset | PubMedBERT question answering | PubMedBERT scores up to 55.84% on the PubMedQA dataset | PubMedBERT | Cluster 1 |
| Deep bidirectional language-knowledge graph pretraining | Michihiro Yasunaga et al. | General Medicine | Diagnostic accuracy | Score in MedQA clinical topics datasets | DRAGON question answering | DRAGON scores up to 47.5% on the MedQA dataset | DRAGON | Cluster 1 |
| Large language models encode clinical knowledge | Karan Singhal et al. | General Medicine | Diagnostic accuracy | Score in MedQA, MedMCQA, PubMedQA, and MMLU clinical topics datasets | Med-PaLM question answering | Med-PaLM scores up to 67.2% on the MedQA dataset | Med-PaLM (Fine tuning) | Cluster 1 |
| Can large language models reason about medical questions? | Valentin Liévin et al | General Medicine | Diagnostic accuracy | Score in MedQA-US Medical Licensing Examination [USMLE], MedMCQA, and PubMedQA | GPT-3.5 vs. Llama 2 | GPT-3.5 scores up to 60.2%, Llama scores up to2-70B 62.5% | GPT-3.5, Llama 2 | Cluster 1 |
| Evaluation and mitigation of cognitive biases in medical language models | Schmidgall S et al. | General Medicine | Diagnostic bias | Score in BiasMedQA dataset | Various LLMs | non-pmc-llama-13b models, gpt-4 shows the smallest drop in average performance (−5.1%), mixtral-8x7b (−7.7%), gpt-3.5 (−17.8%), (−17.9%) and llama-2-70B (−20.1%) | non-pmc-llama-13b models, gpt-4, mixtral-8x7b, gpt-3.5, llama-2-70B | Cluster 1 & Cluster 3 |
| ChatGPT-Generated Differential Diagnosis Lists for Complex Case-Derived Clinical Vignettes: Diagnostic Accuracy Evaluation | Hirosawa, T et al. | General Medicine | Differential diagnosis | Correct diagnosis rate | ChatGPT-3.5 vs. ChatGPT-4 | ChatGPT-4 higher accuracy | ChatGPT-3.5, ChatGPT-4 | Cluster 1 |
| Comparative Study to Evaluate the Accuracy of Differential Diagnosis Lists Generated by Gemini Advanced, Gemini, and Bard for a Case Report Series Analysis: Cross-Sectional Study | Hirosawa, T et al. | General Medicine | Differential diagnosis | Correct diagnosis rate | Gemini Advanced vs. Gemini vs. Bard | Gemini outperformed Bard | Gemini Advanced, Gemini, Bard | Cluster 1 |
| Comparison of emergency medicine specialist, cardiologist, and chat-GPT in electrocardiography assessment | Günay, S et al. | Emergency Medicine/Cardiology | ECG Interpretation | Accuracy in interpreting ECG data | ChatGPT vs. Emergency Medicine Specialists and Cardiologists | ChatGPT outperformed emergency medicine specialists in both everyday and challenging ECG questions | GPT-4 | Cluster 1 & Cluster 2 |
| ChatGPT With GPT-4 Outperforms Emergency Department Physicians in Diagnostic Accuracy: Retrospective Analysis | Hoppe, John Michael et al. | Emergency Medicine | Diagnostic Accuracy | Accuracy | GPT-4 vs. ED Physicians | GPT-4 outperformed ED physicians | GPT-4 | Cluster 1 & Cluster 2 |
| Deep learning-based natural language processing for detecting medical symptoms and histories in emergency patient triage | Lee, S et al. | Emergency Medicine | Symptom and History Detection | Classification Performance | KLUERoBERTa vs. Other models | KLUERoBERTa: F1-score 0.965, AUROC 0.893 | BERT-based | Cluster 1 & Cluster 2 |
| The diagnostic and triage accuracy of the GPT-3 artificial model: an observational study | Levine, DM et al. | Emergency Medicine | Diagnostic and triage accuracy | Correct diagnosis and triage | GPT-3 vs. Laypeople, Physicians | GPT-3 diagnosis accuracy 88%, triage accuracy 70% | GPT-3 | Cluster 1 & Cluster 2 |
| Accuracy Evaluation of GPT-Assisted Differential Diagnosis in Emergency Department | Shah-Mohammadi, F et al. | Emergency Medicine | Differential Diagnosis | Diagnostic Accuracy | GPT-3.5 vs. GPT-4 | GPT-4 slightly outperforms GPT-3.5 in critical categories | GPT-3.5, GPT-4 | Cluster 1 & Cluster 2 |
| Claude 3 Opus and ChatGPT With GPT-4 in Dermoscopic Image Analysis for Melanoma Diagnosis: Comparative Performance Analysis | Liu, X et al. | Dermatology | Diagnostic Performance | Sensitivity, Specificity, Accuracy | Claude 3 Opus vs. ChatGPT | Claude 3 Opus: Sensitivity 54.9%, Specificity 57.14%, Accuracy 56%; ChatGPT: Sensitivity 56.86%, Specificity 38.78%, Accuracy 48% | GPT-4 | Cluster 1 & Cluster 2 |
| Performance of artificial intelligence chatbots in interpreting clinical images of pressure injuries | Shiraishi, M et al. | Dermatology | Image interpretation | Diagnostic Accuracy | ChatGPT-4 Turbo vs. BingAI | ChatGPT-4 Turbo high accuracy (83.0%) | ChatGPT-4 Turbo | Cluster 1 & Cluster 2 |
| Evaluation of the ability of large language models to self-diagnose oral diseases | Zhuang, Shiyang et al. | Dentistry | Self-Diagnosis | Diagnostic Accuracy | - | Potential tool for daily dental care | ChatGPT 3.5 ChatGPT 4.0 PaLM 2 Claude 2 Llama 2 Copilot | Cluster 1 & Cluster 2 & Cluster 3 |
| Assessing the Utility of ChatGPT Throughout the Entire Clinical Workflow: Development and Usability Study | Rao, A et al. | Clinical Workflow | Clinical decision support | Diagnostic Accuracy | ChatGPT vs. Human scorers | 71.7% accuracy in clinical decision-making | GPT-3.5 | Cluster 1 & Cluster 3 |
| Mitigating Cognitive Biases in Clinical Decision-Making Through Multi-Agent Conversations Using Large Language Models: Simulation Study | Ke, YH et al. | Clinical Decision-Making | Cognitive Bias Mitigation | Diagnostic Accuracy | Multi-Agent Framework vs. Human Evaluators | Multi-agent framework accuracy: 76% (top 2 differential diagnoses) | Multi-Agent Framework | Cluster 1 & Cluster 3 |
| In the face of confounders: Atrial fibrillation detection - Practitioners vs. ChatGPT | Avidan, Y et al. | Cardiology | Diagnostic Accuracy | Atrial fibrillation detection accuracy | ChatGPT-4omni vs. Practitioners | ChatGPT suboptimal accuracy with significant under- and over-recognition | ChatGPT-4omni | Cluster 1 & Cluster 2 |
| Evaluating the Performance of Large Language Models in Predicting Diagnostics for Spanish Clinical Cases in Cardiology | Delaunay, Julien et al. | Cardiology | Diagnostic Prediction | Diagnostic Accuracy | State-of-the-art LLMs | Gemini Pro and Mixtral 8x22b performed well | Gemini Pro, Mixtral 8x22b | Cluster 1 & Cluster 2 |
| Generative Pre-trained Transformer 4 analysis of cardiovascular magnetic resonance reports in suspected myocarditis | Kaya, K et al. | Cardiology | Diagnostic accuracy | Sensitivity, specificity, accuracy | GPT-4 vs. Experienced radiologists | GPT-4 accuracy 83%, sensitivity 90%, specificity 78% | GPT-4 | Cluster 1 & Cluster 2 |
| Thinking like a pathologist: Morphologic approach to hepatobiliary tumors by ChatGPT | Laohawetwanit, T et al. | Cancer Pathology | Hepatobiliary tumor diagnosis | Diagnostic accuracy | Morphologic approach vs. Non-morphologic approach | Morphologic approach significantly enhanced accuracy | GPT-4 | Cluster 1 & Cluster 2 & Cluster 3 |
| Accuracy of GPT-4 in histopathological image detection and classification of colorectal adenomas | Laohawetwanit, Thiyaphat et al. | Cancer Pathology | Image Detection & Classification | Diagnostic accuracy; disease classification | - | Sensitivity 74%, Specificity 36% | GPT-4 | Cluster 1 & Cluster 2 |
| Large language models and dermoscopy: Assessing the potential of task-specific GPT-4 vision in diagnosing basal cell carcinoma | Traini DO et al. | Dermatology | Diagnosing basal cell carcinoma | Diagnostic accuracy | - | GPT-4V accuracy of 84.4%, with a sensitivity of 94.0% | GPT-4V | Cluster 1 & Cluster 2 |
