# Supplementary material for: Large Language Models in Medical Diagnostics: Scoping Review With Bibliometric Analysis
Source: J Med Internet Res. 2025 Jun 9;27:e72062. doi: 10.2196/72062 (PMC12186007; doi:10.2196/72062)
Supplement: Multimedia Appendix 3 [file jmir_v27i1e72062_app3.docx]

**Supplementary material 3.** Detail data of LLMs included in accuracy results

| Title | First author | Publication Year | Published Journal | Type of LLM | accuracy /% | Model purpose |
| --- | --- | --- | --- | --- | --- | --- |
| Toward expert-level medical question answering with large language models | Karan Singhal | 2025 | Nature Medicine | Med-PaLM 2 | 86.5 | Medical specific |
| LinkBERT: Pretraining Language Models with Document Links | Michihiro Yasunaga | 2022 | Arxiv | Biolink BERT | 45.1 | Medical specific |
| Domain-Specific Language Model Pretraining for Biomedical Natural Language Processing | Yu Gu | 2021 | ACM Transactions on Computing for Healthcare | PubMedBERT | 38.1 | Medical specific |
| Can large language models reason about medical questions? | Valentin Liévin | 2024 | Patterns | GPT-3.5 | 60.2 | Mixed |
|  |  |  |  | Llama 2-70B | 62.5 | Mixed |
| Capabilities of GPT-4 on Medical Challenge Problems | Harsha Nori | 2023 | Arxiv | GPT-4 | 86.1 | Mixed |
| Deep bidirectional language-knowledge graph pretraining | Michihiro Yasunaga | 2024 | Proceedings of the 36th International Conference on Neural Information Processing Systems | DRAGON | 47.5 | Medical specific |
| Large language models encode clinical knowledge | Karan Singhal | 2023 | Nature | Med-PaLM | 67.2 | Medical specific |
| BioMedLM: A 2.7B Parameter Language Model Trained On Biomedical Text | Elliot Bolten | 2022 | Arxiv | BioMedLM | 50.3 | Medical specific |
| Galactica: a large language model for science | Ross Taylor | 2022 | Arxiv | Glactica | 44.4 | MIxed |
