# Supplementary material for: Large Language Models in Medical Diagnostics: Scoping Review With Bibliometric Analysis
Source: J Med Internet Res. 2025 Jun 9;27:e72062. doi: 10.2196/72062 (PMC12186007; doi:10.2196/72062)
Supplement: Multimedia Appendix 4 [file jmir_v27i1e72062_app4.docx]

**Supplementary material 4.** Characteristic of included clinical trials

| **NCT number** | **First posted date** | **Sponsor** | **Medical speciality** | **Study design** | **Country** | **Phase** | **LLM type** | **Study Title** | **LLMs function** |
| --- | --- | --- | --- | --- | --- | --- | --- | --- | --- |
| NCT06208423 | 2024-01-17 | Stanford University | General medicine | Randomized\|Parallel | Stanford University, United States | Not mentioned | ChatGPT-4 | Physician Reasoning on Management Cases With Large Language Models | Diagnose support |
| NCT06779292 | 2025-01-16 | Capital Medical University | Neurology\|Emergency Medicine | Observational | Xuanwu Hospital, Capital Medical University, China | Not mentioned | Not mentioned | Application of Large Language Models in Emergency Neurology | Direct diagnose |
| NCT05231174 | 2022-02-09 | Sun Yat-sen University | Ophthalmology | Single_group | Zhognshan Ophthalmic Center, Sun Yat-sen University, China | Not mentioned | A self-evaluation tool based on Large Language Model (detail model not mentioned) | Efficacy of Using Large Language Model to Assist in Diabetic Retinopathy Detection | Direct diagnose |
| NCT06865534 | 2025-03-10 | Philipps University Marburg | Oncology | Randomized\|Crossover | Not mentioned | Not mentioned | Local language model (detail model not mentioned) | Large Language Models to Aid Gynecological Oncology Treatment | Diagnose support |
| NCT06157944 | 2023-12-06 | Stanford University | General medicine | Randomized\|Parallel | Stanford University, United States | Not mentioned | ChatGPT-4 | Physician Reasoning on Diagnostic Cases With Large Language Models | Diagnose support |
| NCT06824389 | 2025-02-13 | Zhongshan Ophthalmic Center, Sun Yat-sen University | Ophthalmology | Randomized\|Parallel | Not mentioned | Not mentioned | ChatGPT (detail model not mentioned) | Evaluate the Performance of Large Language Models in Ophthalmologic Patient Consultation | Diagnose support |
| NCT06774612 | 2025-01-14 | Lahore University of Management Sciences | General medicine | Randomized\|Parallel | Lahore University of Management Sciences, Pakistan | Not mentioned | ChatGPT-4o | The Impact of Large Language Models on Diagnostic Reasoning Among Medical Doctors | Diagnose support |
| NCT06486649 | 2024-07-03 | Peking University Third Hospital | Cardiology | Observational | Peking UniversityThird Hospital, China | Not mentioned | Multimodal Large Language Model (detail model not mentioned) | Application of Multimodal Large Language Model in HFpEF | Direct diagnose |
| NCT05816473 | 2023-04-18 | Yale University | Gastroenterology | Parallel | Yale New Haven Hospital, United States | Not mentioned | Not mentioned | Artificial Intelligent Clinical Decision Support System Simulation Center Study for Technology Acceptance | Diagnose support |
| NCT06748170 | 2024-12-27 | Philipps University Marburg | Rheumatology | Randomized\|Parallel | Not mentioned | Not mentioned | ChatGPT (detail model not mentioned) | Al to Improve the Diagnosis of Rare Rheumatic Diseases | Diagnose support |
| NCT06627985 | 2024-10-04 | North Sichuan Medical College | Oncology\| Pulmonology\| Cardiology\| Pulmonology | Observational | The Affiliated Hospital of North Sichuan Medical College, China | Not mentioned | ChatGPT-4o, ChatGPT-4o mini, MedicalGPT, Claude-3.5 Sonnet, Claude 3 Haiku | Multi-Disciplinary Treatment on the Anthropomorphism of Large Language Models | Diagnose support |
| NCT06607822 | 2024-09-23 | The Hong Kong Polytechnic University | Ophthalmology | Randomized\|Parallel | The Hong Kong Polytechnic University, China | Not mentioned | A patient-centered assistant system based on Large-Language Model (detail model not mentioned) | Development and Validation of a Large Language Model-based Myopia Assistant System | Diagnose support |
| NCT06457269 | 2024-06-13 | North Sichuan Medical College | Pulmonology | Randomized\|Crossover | The Affiliated Hospital of North Sichuan Medical College, China | Not mentioned | ChatGPT-3.5, ChatGPT-4.0 , Claude instant, Claude 2,Gemini Pro | Evaluating the Potential of Large Language Models for Respiratory Disease Consultations | Model comparison |
| NCT06002425 | 2023-08-21 | Chinese Academy of Sciences | Oncology\| Gastroenterology | Randomized\|Parallel | United States\|Jiangmen Central Hospital, China\|The Fifth Affiliated Hospital of Sun Yat-sen University, China\|Zhuhai People's Hospital, China\|Peking University Cancer Hospital (Inner Mongolia Campus), China\|University Hospital Magdeburg, Germany\|San Raffaele University Hospital, Italy | Not mentioned | ChatGPT (detail model not mentioned) | Treatment Recommendations for Gastrointestinal Cancers Via Large Language Models | Diagnose support |
| NCT06874647 | 2025-03-13 | Wuhan Union Hospital, China | Radiology | Observational | Not mentioned | Not mentioned | ChatGPT 4o, Deepseek, Janus-Pro | Chest X-Ray Image Diagnosis and Report Generation Dedicated Model Based on Deepseek | Direct diagnose |
| NCT06229379 | 2024-01-29 | Sun Yat-sen University | Ophthalmology | Randomized\|Parallel | Zhongshan Ophthalmic Center, Sun Yat-sen Univerisity, China | Not mentioned | Not mentioned | The Effects of a Large Language Model on Clinical Questioning Skills | Diagnose support |
| NCT06410547 | 2024-05-13 | Charite University, Berlin, Germany | Pulmonology | Randomized\|Parallel | Charité University, Germany | Not mentioned | ChatGPT-4 | Using Large Language Models Such As GPT-4 to Assess Guideline Adherence in Patients with Chronic Obstructive Pulmonary Disease | Diagnose support |
| NCT06510127 | 2024-07-19 | Sun Yat-Sen Memorial Hospital of Sun Yat-Sen University | Oncology | Observational | Sun Yat-Sen Memorial Hospital of Sun Yat-Sen University, China | Not mentioned | Not mentioned | An AI Model Predicts the Efficacy of Neoadjuvant Chemotherapy for Breast Cancer: a Multicenter, Bidirectional Cohort Study | Diagnose support |
| NCT04246346 | 2020-01-29 | Sun Yat-sen University | Ophthalmology | Randomized\|Parallel | Zhognshan Ophthalmic Center, Sun Yat-sen University, China | Not mentioned | An interactive chatbot for patient decision aid (detail model not mentioned) | Effectiveness of Using Interactive Consulting System to Enhance Informed Choice | Diagnose support |
| NCT06856668 | 2025-03-04 | Northwestern University | Psychiatry | Randomized\|Parallel | Not mentioned | Not mentioned | Not mentioned | A Megastudy of Single-Session Interventions for Depression | Direct diagnose/intervention |
| NCT05789901 | 2023-03-29 | McGill University Health Centre/Research Institute of the McGill University Health Centre | Oncology\| Emergency Medicine | Non_randomized\|Parallel | Centre hospitalier de l'Université de Montréal (CHUM), Canada\|McGill University Health Centre (MUHC), Canada | Not mentioned | MARVIN, MARVIN-Pharma, MARVINA, MARVIN-CHAMP | The MARVIN Chatbots to Provide Information for Different Health Conditions | Diagnose support |
| NCT06710860 | 2024-11-29 | Sun Yat-sen University | Oncology\| Gastroenterology | Randomized\|Parallel | Sun Yat-sen University Cancer Center, China\|The Third Affiliated Hospital of Sun Yat-sen University, China\|The Sixth Affiliated Hospital of Sun Yat-sen University, China | Not mentioned | Not mentioned | AI Assistant for Enhancing Colorectal Cancer Screening Among First-degree Relatives of Patients: a Randomized Trial | Diagnose support |
| NCT06334796 | 2024-03-28 | Fundación para la Lucha contra las Enfermedades Neurológicas de la Infancia | Neurology\| Neurosurgery | Non_randomized\|Single_group | Fleni, Argentina | EARLY_PHASE1 | Self develop AI assistant | Artificial Intelligence-powered Virtual Assistant for Emergency Triage in Neurology | Direct diagnose |
